# Design of Pd-based pseudo-binary alloy catalysts for highly active and selective NO reduction†
†Electronic supplementary information (ESI) available: Details of characterization, kinetic analysis, and DFT calculations. See DOI: 10.1039/c8sc05496g


**DOI:** 10.1039/c8sc05496g

**Published:** 2019-03-04

**Authors:** Jaewan Jeon, Ken-ichi Kon, Takashi Toyao, Ken-ichi Shimizu, Shinya Furukawa

**Affiliations:** a Institute for Catalysis , Hokkaido University , N21, W10 , Sapporo 001-0021 , Japan . Email: furukawa@cat.hokudai.ac.jp ; Fax: +81-11-706-9163; b Elements Strategy Initiative for Catalysts and Batteries , Kyoto University , Katsura , Kyoto 615-8520 , Japan

## Abstract

The development of Pd-based alloy catalysts for highly active and selective reduction of NO by CO was investigated. A survey of Pd-based bimetallic catalysts (PdM/Al_2_O_3_: M = Cu, In, Pb, Sn, and Zn) revealed that the PdIn/Al_2_O_3_ catalyst displayed excellent N_2_ selectivity even at low temperatures (100% at 200 °C). The catalytic activity of PdIn was further improved by substituting a part of In with Cu, where a Pd(In_1–*x*_Cu_*x*_) pseudo-binary alloy structure was formed. The optimized catalyst, namely, Pd(In_0.33_Cu_0.67_)/Al_2_O_3_, facilitated the complete conversion of NO to N_2_ (100% yield) even at 200 °C and higher, which has never been achieved using metallic catalysts. The formation of the pseudo-binary alloy structure was confirmed by the combination of HAADF-STEM-EDS, EXAFS, and CO-FT-IR analyses. A detailed mechanistic study based on kinetic analysis, operando XAFS, and DFT calculations revealed the roles of In and Cu in the significant enhancement of catalytic performance: (1) N_2_O adsorption and decomposition (N_2_O → N_2_ + O) were drastically enhanced by In, thus resulting in high N_2_ selectivity; (2) CO oxidation was promoted by In, thus leading to enhanced low-temperature activity; and (3) Cu substitution improved NO adsorption and dissociation (NO → N + O), thus resulting in the promotion of high-temperature activity.

## Abstract 2

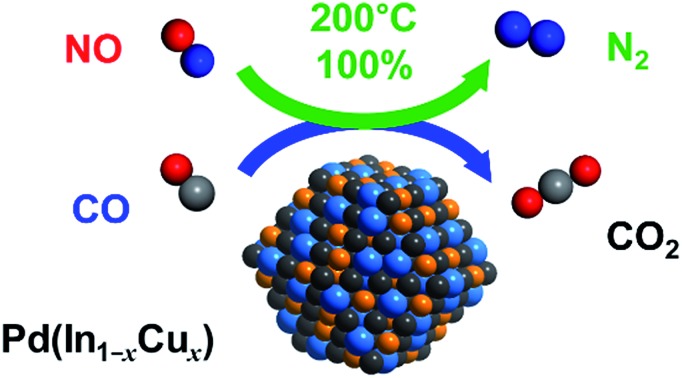
Drastic tuning of NO reduction activity and N_2_ selectivity based on the catalyst design with pseudo-binary alloy structures.

## 1. Introduction

Reactions of nitric oxide (NO) have received an increasing amount of interest among researchers for human health,^1^ bioinorganic,^2^ industrial,^3^ and environmental chemistry applications.^4^ Specifically, NO removal has long been studied as an indispensable process for exhaust gas purification.^5^ Platinum group metals (PGMs) such as Pt, Rh, and Pd are known as efficient catalysts for NO reduction with CO,^6^,^7^ H_2_,^8^ NH_3_,^9^ and hydrocarbons^10^ as reductants. Attention has been increasingly focused on the use of Pd because of its prominent oxidation activity for hydrocarbons and CO,^11^ and its excellent thermal stability.^12^ However, controlling the selectivity of NO reduction to N_2_ remains a significant challenge because significant amounts of undesired by-products, such as N_2_O, which is a powerful greenhouse gas, are particularly evolved when CO is used as a reductant.^6^ To the best of our knowledge, no metallic catalyst that shows high NO reduction ability without N_2_O emission even at low temperatures (<250 °C) has been reported in the literature. In this context, a drastic improvement in the nature of metallic catalysts for this reaction is an important measure that will address a number of global environmental issues and fundamental chemistry. The mechanism of NO reduction on the surface of PGMs has been extensively studied, and the following is generally accepted:^5^,^10^,^13^ (1) NO → N + O (NO dissociation), (2) N + N → N_2_ (N_2_ formation), (3) N + NO → N_2_O (side reaction), (4) N_2_O → N_2_ + O (N_2_O decomposition), and (5) O + R → RO (oxygen consumption, R = CO or H_2_). Hence, the suppression of (3) and/or the promotion of (4) are required to develop a highly selective NO reduction system. Theoretical approaches have also been studied and have predicted that a stepped (211) surface of PGMs was capable of suppressing (3).^14^,^15^ However, it is difficult to realize a high fraction of such a metastable surface. Therefore, the appropriate design and construction of an ideal reaction environment using metallic materials are necessary.

Intermetallic compounds, which are ordered alloys typically composed of certain metal elements that are separated from one another in the periodic table, can be attractive inorganic materials for sophisticated catalyst design.^16^ An appropriate combination of two or three metal elements would provide significantly modified electronic and geometric structures for active sites and a highly ordered reaction environment for NO reduction.^16^ Indeed, a recent theoretical prediction suggested that a Pd–Ti bimetallic surface was capable of NO reduction by CO without emission of N_2_O even at room temperature and higher.^17^ Although this is an attractive prediction, there remains another task of retaining the metallic state of electropositive Ti at the surface, which is oxidized under a ppb-level oxygen atmosphere.^18^ Therefore, in view of practical use, it is also important to choose counterpart elements that have appropriate redox properties.

In this study, we prepared a series of Pd-based intermetallic compound catalysts by using an alumina support (PdM/Al_2_O_3_, M = Cu, Fe, In, Pb, and Zn) and tested them for selective NO reduction with CO as a reductant. The effect of the second metal on improved selectivity was deeply investigated using characterization techniques, kinetic studies, and DFT calculations. Furthermore, the selective bimetallic catalyst was further improved by constructing pseudo-binary alloy structures to develop an innovatively efficient catalytic system. We report a highly active and selective NO reduction system that is based on a novel concept of catalyst design and the fundamental aspects governing catalytic performance in such NO reduction systems.

## 2. Methods

### 2.1. Catalyst preparation

Boehmite (γ-AlOOH) was supplied by SASOL chemicals. γ-Al_2_O_3_ was prepared by the calcination of boehmite at 900 °C for 3 h. A series of Pd-based catalysts (PdM/Al_2_O_3_, M = Cu, In, Zn, Sn and Pb) were prepared by a co-impregnation method using an excess amount of water (*ca.* 25 ml of ion exchanged water per g of support). The γ-Al_2_O_3_ support was added to a vigorously stirred mixed aqueous solution of Pd(NH_3_)_2_(NO_2_)_2_ and a second metal salt (Cu(NO_3_)_2_·3H_2_O (Sigma-Aldrich, 99%, In(NO_3_)_3_·*n*H_2_O (Kanto, 99%), Zn(NO_3_)_2_·6H_2_O (Kanto, 99%), SnCl_2_ (Wako, 99.9%) and Pb(NO_3_)_2_ (Wako, 99.5%)), followed by stirring for 2 h. The mixture was dried under a reduced pressure at 50 °C, followed by reduction under flowing H_2_ (30 ml min^–1^) at 400 °C for 1 h. The metal loading and the atomic ratio of Pd/M were adjusted to 3 wt% and 1 (M = Cu, In, and Zn) and 3 (M = Pb and Sn), respectively. For trimetallic catalysts, the metal loading of Pd was also adjusted to 3 wt% and the atomic ratio of Pd/(M + N) was one. The as-impregnated catalysts were reduced under flowing H_2_ at 400 °C (M = Cu, In, and Zn) or 600 °C (M = Pb and Sn) for 1 h.

### 2.2. Catalytic reactions

The catalyst (0.015 g) diluted with quartz sand (1.985 g, Miyazaki Chemical 99.9%) was treated under flowing hydrogen (50 ml min^–1^) at 400 °C for 0.5 h prior to the catalytic reactions. NO reduction by CO was performed in a fixed-bed continuous flow system by feeding NO (0.5%), CO (0.5%), and He (balance) with a total flow rate of 96 ml min^–1^ (GHSV = 240 000 h^–1^). The corresponding N_2_O reduction was also performed in a similar fashion with an N_2_O concentration of 0.5%. The gas phase was analyzed using an online thermal conductivity detection gas chromatograph (Shimazu GC-8A, column: SHINWA SHINCARBON ST) located downstream. A kinetic study was performed by changing the concentration of NO and CO between 0.3–0.6% with that of the counterpart fixed at 0.5%. The reaction temperature was maintained at 150 °C so that NO conversion did not exceed 30%, and the reaction rate (mol s^–1^ mol_Pd_^–1^) was calculated on the basis of NO conversion.

### 2.3. Characterization

The crystal structure of the prepared catalyst was examined by powder X-ray diffraction (XRD) on a Rigaku MiniFlex II/AP diffractometer with Cu Kα radiation. High angle annular dark field scanning TEM microscopy (HAADF-STEM) was carried out using a JEOL JEM-ARM200 M microscope equipped with an energy dispersive X-ray (EDX) analyzer (EX24221M1G5T). The STEM analysis was performed at an accelerating voltage of 200 kV. To prepare the TEM specimen, all samples were sonicated in ethanol and then dispersed on a Mo grid supported by an ultrathin carbon film.

CO pulse chemisorption was performed using a BELCAT II (Microtrac BEL) to estimate the Pd dispersion of the prepared catalysts. Prior to chemisorption, the catalyst was pretreated under a 5% H_2_/Ar flow (40 ml min^–1^) at 400 °C for 0.5 h. After the reduction pretreatment, He was introduced at the same temperature for 10 min to remove the chemisorbed hydrogen, followed by cooling to room temperature. A 10% CO/He pulse was introduced into the reactor, and the supplied CO flow was quantified downstream using a thermal conductivity detector. For example, Pd dispersion for PdIn/Al_2_O_3_ (Pd: 3 wt%) was estimated as 11.0% (the results of XRD, STEM, and CO pulse adsorption for representative Pd-based catalysts are summarized in Table S1†).

The Fourier-transformed infrared (FT-IR) spectra of adsorbed CO were obtained with a JASCO FTIR-4200 spectrometer equipped with an MCT detector in the transmission mode (resolution 4 cm^–1^). The samples were prepared as self-supporting wafers (2.0 cm diameter. <0.5 mm thickness) and were placed inside an IR cell with CaF_2_ windows. A custom glass manifold was connected to the cell to control the gas for pretreatment and the amount of CO introduced. The cell was first purged with He, and the sample was reduced under flowing hydrogen (50 ml min^–1^) at 400 °C for 30 min. After reduction, the wafer was cooled to 40 °C under He flow. The wafer was exposed to CO (0.5%) and He (balance) with a total flow rate of 50 ml min^–1^ for 20 min. After the CO exposure, He was flowed for 5 min to remove the gas phase and weakly adsorbed CO, followed by IR spectra measurements.

X-ray absorption fine structure (XAFS) spectra were recorded on the BL14B2 and BL01B1 stations at SPring-8 of the Japan Synchrotron Radiation Research Institute. A Si(311) double-crystal monochromator was used. Energy calibration was performed using Pd foil. The spectra were recorded at the edges of Pd K, In K, and Cu K in a transmission mode at room temperature. For static measurements, the pelletized sample was prereduced with H_2_ at 400 °C for 0.5 h, and then sealed in a plastic pack under a N_2_ atmosphere together with an ISO A500-HS oxygen absorber (Fe powder). For operando analysis, the pelletized sample (600 mg of 1.0 wt% Pd/Al_2_O_3_ or PdIn/Al_2_O_3_: 200 mg 10*φ* disk × 3) was introduced into a quartz cell equipped with Kapton film windows and gas lines connected to a high sampling rate TCD GC (490 Micro GC, Agilent Technologies Inc.) downstream for the quantification of CO, N_2_, CO_2_, and N_2_O. The catalyst in the cell was reduced by flowing 10% H_2_/He at 400 °C for 15 min, followed by cooling to 200 °C under He purge. Thereafter, NO + CO, NO, and CO (0.5% for each with He balance for a total of 800 ml min^–1^; GHSV = 200 000 h^–1^) were fed into the cell for 20 min for each gas with 10 min intervals of He purging. The catalyst and products in the gas phase were monitored simultaneously by XAFS and GC, respectively. The obtained XAFS spectra were analyzed using Athena and Artemis software ver. 0.9.25 included in the Demeter package.^19^ The obtained XANES spectra were fitted by a linear combination of those of the reduced catalysts and metal oxides (PdO or In_2_O_3_) within the range of –30 to +50 eV toward *E*_0_. Each linear combination fitting was successfully accomplished with an *R* factor less than 0.01. The Fourier transformation of the *k*^2^-weighted EXAFS from *k* space to *R* space was performed over a *k* range of 3.0–15 Å^–1^. A part of the Fourier-transformed EXAFS in the *R* range of 1.2–3.0 Å was inversely Fourier transformed, followed by an analysis using a usual curve fitting method in a *k* range of 3–15 Å^–1^. The back-scattering amplitude or phase shift parameters were simulated with FEFF 6L and used to perform the curve fitting procedure. The amplitude reduction factors (*S*_0_^2^) were determined by fitting the spectra of reference samples (Pd black: 0.761, PdO: 0.827, In_2_O_3_: 1.112, Cu foil: 0.899, CuO: 0.906) and then they were used for the fitting of other EXAFS spectra.

### 2.4. Computational details

Periodic DFT calculations were performed using the CASTEP code^20^ with Vanderbilt-type ultrasoft pseudopotentials^21^ and the Perdew–Burke–Ernzerhof exchange–correlation functional based on the generalized gradient approximation.^22^ The plane-wave basis set was truncated at a kinetic energy of 360 eV. A Fermi smearing of 0.1 eV was utilized. Dispersion correlations were considered using the Tkatchenko–Scheffler method with a scaling coefficient of *s*_R_ = 0.94 and a damping parameter of *d* = 20.^23^ The reciprocal space was sampled using a *k*-point mesh with a spacing of typically 0.04 Å^–1^, as generated by the Monkhorst–Pack scheme.^24^ Geometry optimizations and transition state (TS) searches were performed on supercell structures using periodic boundary conditions. The surfaces were modeled using metallic slabs with a thickness of six atomic layers with 13 Å of vacuum spacing. The unit cells were (2 × 2) for Pd(111) and PdIn(120), (2 × 3) for Pd(511), (3 × 2) for PdIn(110), and (3 × 3) for Pd(100). We chose PdIn(110) as the most stable and dominant surface as reported in some literature.^25^,^26^ PdIn(120) was modeled as the stepped PdIn(110). Pd(511) was reported by Sautet *et al.*^27^ as the most active stepped surface for NO dissociation. Geometry optimizations were performed using the Broyden–Fletcher–Goldfarb–Shanno (BFGS) algorithm.^28^ The unit cell size of the bulk material was firstly optimized, followed by modeling the slab structure and surface relaxation with the size of the supercell fixed. The convergence criteria for structural optimization and energy calculations were set to (a) an SCF tolerance of 1.0 × 10^–6^ eV per atom, (b) an energy tolerance of 1.0 × 10^–5^ eV per atom, (c) a maximum force tolerance of 0.05 eV Å^–1^, and (d) a maximum displacement tolerance of 1.0 × 10^–3^ Å.

The adsorption energy was defined as follows: *E*_ad_ = *E*_A–S_ – (*E*_S_ + *E*_A_), where *E*_A–S_ is the energy of the slab together with the adsorbate, *E*_A_ is the total energy of the free adsorbate, and *E*_S_ is the total energy of the bare slab. The adsorption energy for an oxygen-preadsorbed slab was calculated using *E*_SH_, which is the total energy of the oxygen-adsorbed slab, instead of using *E*_S_.

The TS search was performed using the complete linear synchronous transit/quadratic synchronous transit (LST/QST) method.^29^,^30^ Linear synchronous transit maximization was performed, followed by energy minimization in the directions conjugating to the reaction pathway. The approximated TS was used to perform QST maximization with conjugate gradient minimization refinements. This cycle was repeated until a stationary point was found. Convergence criteria for the TS calculations were set to root-mean-square forces on an atom tolerance of 0.10 eV Å^–1^.

## 3. Results

### 3.1. Bimetallic system

**Fig. 1:**
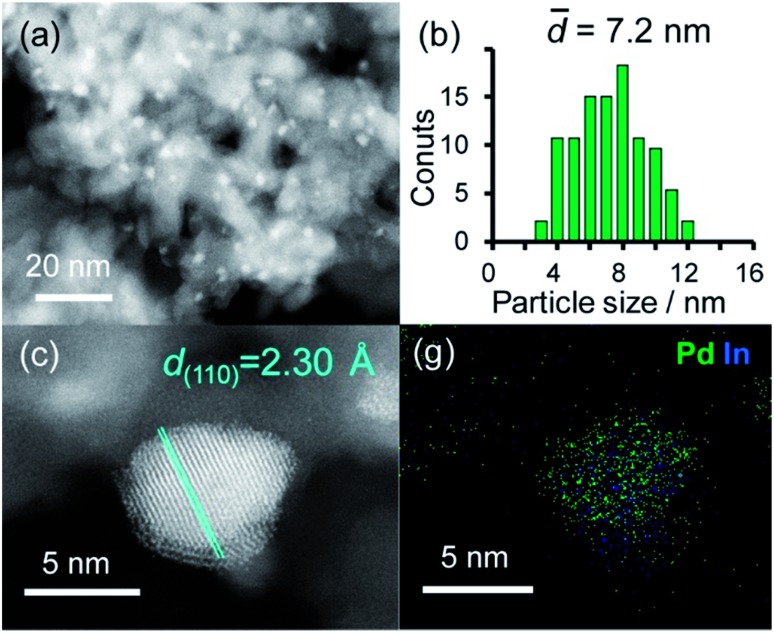
(a) HAADF-STEM image of PdIn/Al_2_O_3_ and (b) size distribution of the nanoparticles. (c) High-resolution STEM image of a single nanoparticle and (d) elemental maps of Pd and In in the nanoparticle acquired using EDX.

A series of Pd-based intermetallic catalysts was prepared using a co-impregnation method with γ-Al_2_O_3_ as the support (PdM/Al_2_O_3_, M = Cu, In, Zn, Sn and Pb). The crystalline phases of the prepared catalysts were analyzed using XRD (Fig. S1†). For each catalyst, the diffraction peak assigned to the desired intermetallic phase was observed in addition to the peaks from the γ-Al_2_O_3_ support. Fig. 1a and b show the HAADF-STEM image of PdIn/Al_2_O_3_ and the size distribution of the nanoparticles, respectively.

Particle sizes ranged from 3 to 12 nm with a volume weighted average of 7.2 nm, which is consistent with the crystallite size estimated from the Scherrer equation (6.6 nm). Fig. 1c shows the high-resolution image of a single nanoparticle. Lattice fringes with 2.30 Å spacing were clearly observed, and this finding is finely consistent with the interplanar distance of the PdIn(110) plane (2.30 Å).^31^ The elemental maps of Pd and In that were acquired using the EDX analysis of this field revealed that the Pd and In atoms comprising the nanoparticles were homogeneously dispersed (Fig. 1d).

**Fig. 2:**
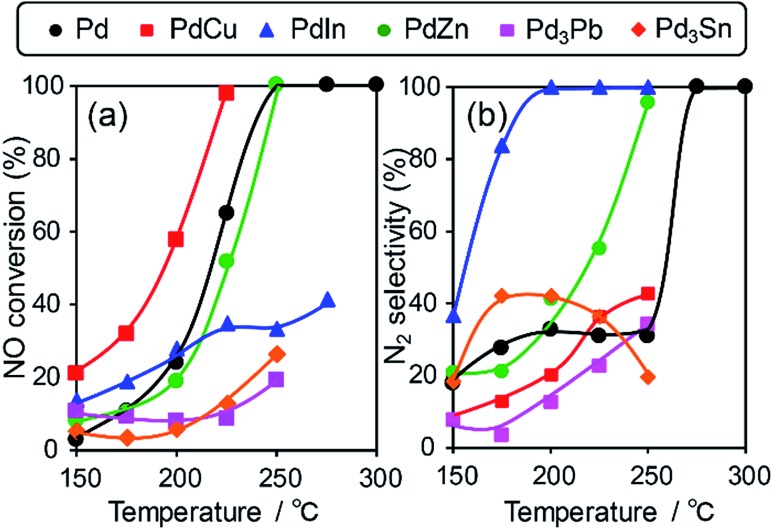
(a) NO conversion and (b) N_2_ selectivity in NO reduction by CO using Pd based catalysts.

We then tested the catalytic performance of Pd-based catalysts (PdM/Al_2_O_3_; M = Cu, In, Zn, Pb, and Sn, Pd: 3 wt%) in NO reduction by CO. Fig. 2 shows the temperature dependence of (a) NO conversion and (b) N_2_ selectivity obtained using these catalysts.

Note that harsh conditions (NO, CO = 5000 ppm, GHSV = 240 000 h^–1^) were employed so that the difference in catalytic performances became obvious. Pd/Al_2_O_3_ showed a light-off temperature of *ca.* 220 °C, and full NO conversion was obtained at 250 °C. The N_2_ selectivity was approximately 30% below 250 °C, which was indicative of the significant formation of N_2_O (∼70%) and the intrinsic low N_2_ selectivity of Pd for NO reduction to N_2_ at low temperatures. PdCu and PdZn showed similar light-off behavior. For Pd-based bimetallic catalysts, N_2_ selectivity monotonically increased up to 100% as the reaction temperature increased. In contrast, N_2_ selectivity in the low-temperature region (250 °C) was quite different depending on the second metal (PdIn ≫ PdZn > PdCu > Pd > Pd_3_Pb). PdIn exhibited very high selectivities over a wide range of temperatures (100% even at 200 °C and higher); to the best of our knowledge, this finding has never been reported for this reaction when using metal catalysts. Therefore, the formation of intermetallic phases drastically modified the N_2_ selectivity in NO reduction by CO and allowed highly selective conversion.

However, the catalytic activity of PdIn was not sufficient, particularly in the high-temperature region (Fig. 2a). For better NO_*x*_ removal performance, both N_2_ selectivity and NO conversion should also be enhanced. We then focused on improving the catalytic activity of the PdIn catalyst. We first tried to use a series of Pd-rich Pd–In catalysts (Pd/In = 1, 2, 3, and 5.67) to increase the reaction rate. However, NO conversion did not increase and N_2_ selectivity was significantly decreased as the Pd/In ratio increased (Fig. S2†). Therefore, a conventional method for changing the Pd/In ratio did not effectively improve catalytic performance. In general, because the structure and electronic state of intermetallic compounds are determined depending on the element and composition ratio, these factors cannot be tuned flexibly and independently. Therefore, an appropriate methodology that finely tunes the nature of intermetallic catalysts must be developed.

### 3.2. Trimetallic system

**Scheme 1:**
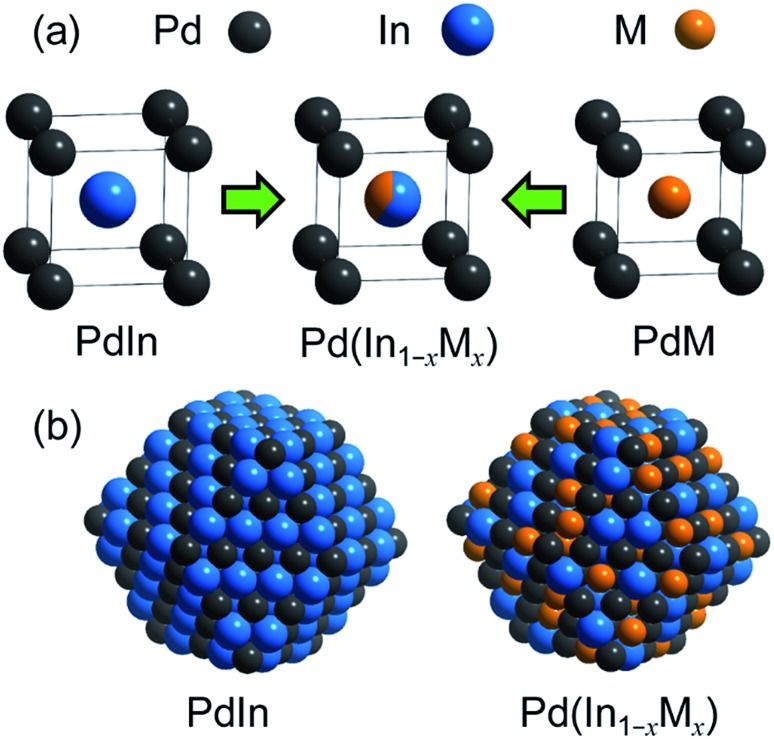
(a) Concept of the PdIn-based pseudo-binary alloy having a CsCl-type structure with the substitution of In by M. (b) Models of nanoparticulate PdIn and Pd(In_1–*x*_M_*x*_) with a rhombohedral structure surrounded by {110} surfaces. A part of In is replaced by M without modification of Pd.

For catalyst design with such an ideal concept, we have focused on a pseudo-binary alloy structure^32^–^34^ of Pd(In_1–*x*_M_*x*_) using a third metal element M (Scheme 1).

**Fig. 3:**
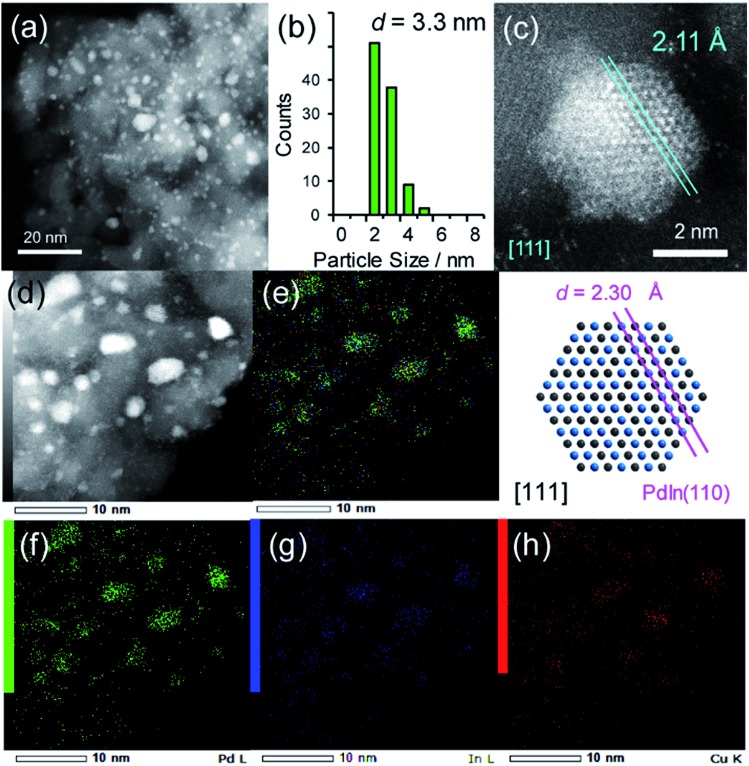
(a) HAADF-STEM image of Pd–In–Cu/Al_2_O_3_ (Pd : In : Cu = 3 : 1 : 2) and (b) size distribution of the nanoparticle. (c) High-resolution STEM image of a single nanoparticle (top) and rhombohedral crystal of PdIn viewed along the [111] direction (bottom). (d) HAADF-STEM image of Pd–In–Cu/Al_2_O_3_ and elemental maps of (e) the Pd + In + Cu overlayer, (f) Pd, (g) In, and (h) Cu acquired using EDX.

This compound retains the CsCl-type structure of intermetallic PdIn with a part of In replaced by M, thus enabling the gradual modification of the In/M ratio and catalytic properties. This structure can also be regarded a solid solution alloy of CsCl-type PdIn and PdM, namely, (PdIn)_1–*x*_(PdM)_*x*_.^32^ Several metal elements such as Ti,^35^ Mn,^36^ Fe,^37^ Cu,^38^ Zn,^39^ and Ga,^40^ which form intermetallic phases with bcc structures, are potential candidates for the third metal M. Here, we first chose Cu as the third metal M because PdCu exhibited a high catalytic activity (Fig. 2a). Several Pd–In–Cu/Al_2_O_3_ catalysts with various In/Cu ratios were prepared using the co-impregnation method as used for bimetallic materials. Fig. 3a shows the HAADF-STEM images of a Pd–In–Cu/Al_2_O_3_ (Pd : In : Cu = 3 : 1 : 2) catalyst.

The size distribution of the nanoparticles shifted to a smaller region (2–5 nm, Fig. 3b) than that of PdIn/Al_2_O_3_. Similar to the observation for the Pd–Cu catalyst (Fig. S1†), the presence of Cu might inhibit the aggregation of the metal nanoparticles during catalyst preparation. The EDX mapping for some nanoparticles showed that Pd, In, and Cu were homogeneously dispersed and constituted the nanoparticles (Fig. 3d–h). A high-resolution image of a single nanoparticle displayed the atomic arrangement of a CsCl-type crystal viewed along the [111] direction with an interplanar distance of 2.11 Å for {110} planes (Fig. 3c). Note that this structure cannot be assigned to any fcc structure because the corresponding atomic arrangement with six-fold symmetry for an fcc crystal appears with significantly shorter interplanar distances (*e.g.*, 1.38 Å for Pd(220)). The observed interplanar distance is close to but slightly shorter than that of PdIn(110), namely 2.30 Å,^31^ suggesting the shrinkage of the PdIn crystal lattice by the substitution of In with Cu. By applying Vegard's law on the basis of their atomic radii^41^ and ratios (Pd: 1.373 Å, In: 1.660 Å, Cu: 1.276 Å, 3 : 1 : 2), it is possible to estimate the theoretical shrinkage of the crystal lattice to be 8.4% (see ESI for details, Fig. S3†). This value agrees with the experimental shrinkage observed in Fig. 3c (8.2%) and supports the formation of a Pd(In_0.33_Cu_0.67_) pseudo-binary alloy structure.

We also performed EXAFS analysis for PdIn and Pd–In–Cu catalysts to obtain structural information for the entire catalyst. Fig. S4† shows the raw EXAFS oscillations, their Fourier-transforms, and the corresponding curve fits and Table 1 summarizes the curve fitting results.

**Table 1:** Summary of EXAFS curve fitting for Pd-based catalysts

Sample	Edge	Shell	*N*	*R* (Å)	Δ*E* (eV)	*σ* ^2^ (Å^2^)	*R*-Factor
Pd black	Pd K	Pd–Pd	12 (fix)	2.74 ± 0.00	0.0 ± 0.3	0.006	0.002
Pd/Al_2_O_3_	Pd K	Pd–O	0.7 ± 0.2	2.02 ± 0.02	6.4 ± 3.2	0.004	0.002
		Pd–Pd	7.1 ± 0.3	2.73 ± 0.00	0.0 ± 0.4	0.009	
PdIn/Al_2_O_3_	Pd K	Pd–In	6.6 ± 0.3	2.73 ± 0.00	0.0 ± 0.3	0.012	0.012
	In K	In–O	2.5 ± 0.3	2.14 ± 0.01	2.3 ± 1.5	0.010	0.014
		In–Pd	5.7 ± 0.6	2.74 ± 0.01	0.0 ± 0.7	0.014	
Pd–In–Cu/Al_2_O_3_	Pd K	Pd–Cu	3.0 ± 0.3	2.60 ± 0.01	1.8 ± 1.6	0.006	0.002
(Pd : In : Cu = 3 : 1 : 2)		Pd–In	5.8 ± 0.5	2.70 ± 0.01	–1.3 ± 0.7	0.013	
	In K	In–O	2.6 ± 0.3	2.13 ± 0.01	4.1 ± 1.5	0.010	0.010
		In–Pd	5.8 ± 0.5	2.70 + 0.00	0.0 ± 0.6	0.010	
	Cu K	Cu–O	1.0 ± 0.2	1.93 ± 0.02	5.8 ± 2.3	0.006	0.006
		Cu–Cu	2.2 ± 1.6	2.58 ± 0.08	–1.0 ± 9.5	0.013	
		Cu–Pd	3.1 ± 0.9	2.59 ± 0.02	–3.2 ± 0.7	0.009	

For the PdIn catalyst, the Pd–In bond lengths observed from the edges of Pd K and In K agreed with each other within the error bar. For the Pd–In–Cu catalyst, two different scatterings assigned to Pd–Cu and Pd–In were observed in the Pd K-edge EXAFS. The corresponding Cu–Pd and In–Pd shells were also confirmed in the In K- and Cu K-edge EXAFS, respectively, in good agreement with the coordination numbers and bond lengths. Assuming In–Cu scattering in the In K edge instead of and in addition to In–Pd scattering did not result in good fittings (*R* > 0.1 and negative N for In–Cu, respectively, data not shown). This suggests that (1) In atoms in the alloy phase are surrounded by Pd; hence (2) Pd is not substituted by Cu. These results strongly suggest the formation of a Pd(In_1–*x*_Cu_*x*_) pseudo-binary alloy structure. Moreover, the Pd–In interatomic distance of the Pd–In–Cu catalyst (2.70 Å) was slightly shorter than that of PdIn (2.73 Å). This is consistent with the shrinkage of the PdIn lattice by the incorporation of Cu (Fig. 3c) and excludes the possibility that the catalyst is a physical mixture of PdIn and PdCu phases. For Cu K-edge EXAFS, the small contributions of Cu–Cu scattering were also observed in addition to Cu–Pd scattering. Considering (2), it is likely that there remains a small amount of monometallic Cu that did not participate in the formation of alloy species, which cannot be detected in Pd K and In K edges. The coordination number of the Pd–Cu shell in Pd–In–Cu was smaller than that of the Pd–In shell despite the higher feeding ratio of Cu than In probably because of the presence of monometallic Cu and/or the inhomogeneous distribution of Cu in Pd(In_1–*x*_Cu_*x*_) (*e.g.*, Cu-rich shell and In-rich core). Small contributions of M–O scatterings were also observed for all samples probably because of the interaction between the metal nanoparticles and the Al_2_O_3_ support and/or the aerobic oxidation of the surface region by trace amounts of oxygen impurities.

**Fig. 4:**
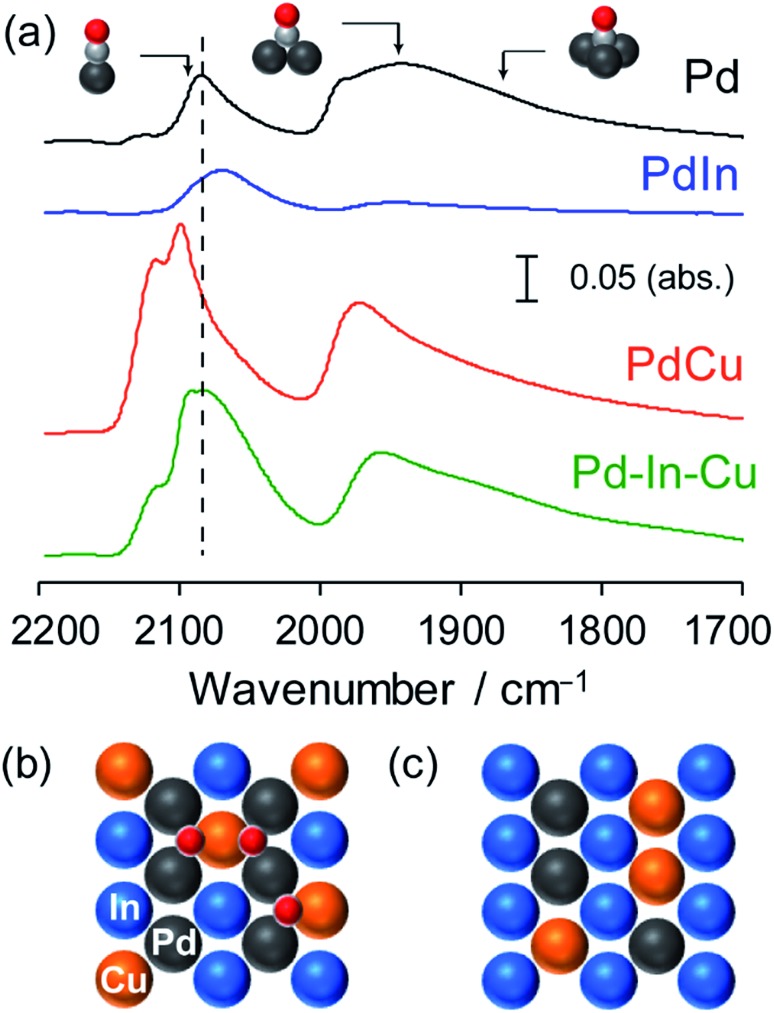
(a) FT-IR spectra of CO adsorbed on Pd/Al_2_O_3_, PdIn/Al_2_O_3_, PdCu/Al_2_O_3_ and Pd–In–Cu/Al_2_O_3_ (Pd : In : Cu = 3 : 1 : 2) catalysts. Atomic arrangement of the most stable (110) surface of Pd(In_1–*x*_Cu_*x*_) assuming (b) In–Cu and (c) Pd–Cu replacement. Pd_2_Cu hollow sites are formed in (b).

FT-IR analysis was also performed for the Pd-based catalysts by using CO adsorption to obtain information about the electronic and geometric environment at the surface (Fig. 4). Pd/Al_2_O_3_ showed three different features assigned to the stretching vibration of CO adsorbed on top (2086 cm^–1^), bridge (∼1950 cm^–1^), and threefold hollow (∼1860 cm^–1^) sites of Pd.^42^ For PdIn/Al_2_O_3_, the peak position of linear CO was red-shifted (2068 cm^–1^) and the peak corresponding to the bridge CO almost vanished. This indicates that (1) the Pd in PdIn is electron enriched by In, (2) the Pd–Pd ensembles are completely diluted by In, and (3) the catalyst surface is also of intermetallic PdIn. A different trend was observed for PdCu/Al_2_O_3_, wherein the peak of the on-top CO was blue-shifted (2099 and 2118 cm^–1^) from that of Pd and a part of bridge CO remained. The observed two on-top CO molecules could be assigned to those on Pd (2099 cm^–1^) and Cu (2118 cm^–1^) sites.

Although CO adsorption on metallic Cu is generally accepted to be very weak, our recent report revealed that CO can be adsorbed on the metallic Cu sites of intermetallic PtCu, where linear CO on Pt and Cu appeared at 2050 and 2120 cm^–1^, respectively.^43^ Considering that Pd–Pd ensembles are sufficiently diluted by Cu, the appearance of the threefold CO suggests that Pd_2_Cu hollows work as adsorption sites of CO. Recent experimental and theoretical studies suggested that CO adsorption on the Pd_2_Cu hollow site was reasonable.^44^,^45^ Pd–In–Cu/Al_2_O_3_ showed an intermediate feature between PdIn and PdCu, that is, linear CO of which the peak position was close to that of Pd and small amounts of bridge and threefold CO remained. This indicates that the electronic effects of In and Cu on Pd cancelled each other out and that Pd, In, and Cu are all present at the surface. The observation of threefold CO is consistent with the pseudo-binary alloy structure with In–Cu substitution, where the Pd_2_Cu hollow site is formed on the most stable (110) surface (Fig. 4b). If Cu is substituted exclusively with Pd, Pd-less ensembles such as PdInCu or Cu_2_In hollow sites are only allowed at the (110) surface (Fig. 4c), resulting in no bridge or threefold CO. Thus, the FT-IR study supported the proposition that the intermetallic and pseudo-binary alloy structures were also formed at the catalyst surface.

**Fig. 5:**
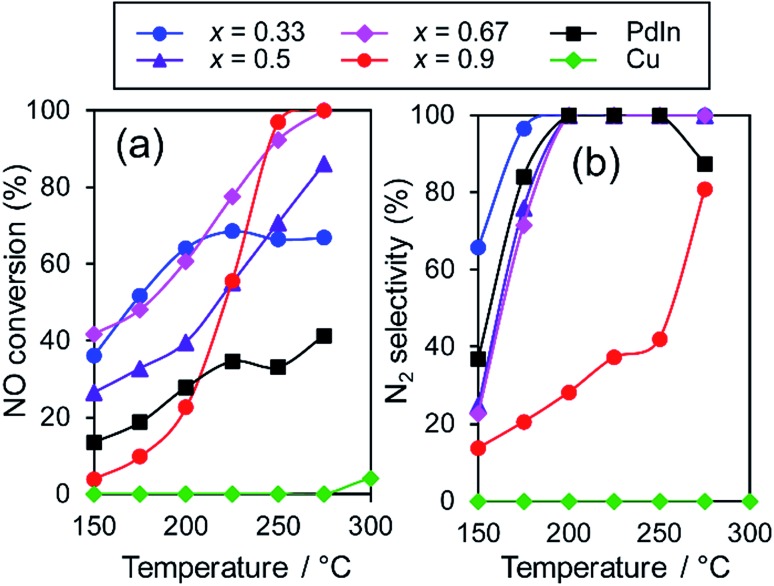
Temperature dependence of NO conversion and N_2_ selectivity in NO reduction by CO over various Pd–In–Cu/Al_2_O_3_ (Pd : In : Cu = 1 : 1–*x* : *x*) catalysts.

The prepared trimetallic catalysts with various Cu contents (Pd–In–Cu/Al_2_O_3_, Pd : In : Cu = 1 : 1 – *x* : *x*, *x* = 0.33, 0.5, 0.67, and 0.9) were tested in NO reduction by CO to evaluate the effect of Cu substitution. Fig. 5 shows the temperature dependence of the NO conversion and N_2_ selectivity obtained with these catalysts. In most cases, NO conversion was drastically increased without lowering the N_2_ selectivity by Cu substitution. The In-rich catalyst (*x* = 0.33) showed a temperature-dependence similar to that of PdIn (*x* = 0, without light-off behavior), thus resulting in NO conversions plateauing in the high-temperature region (>225 °C).

**Fig. 6:**
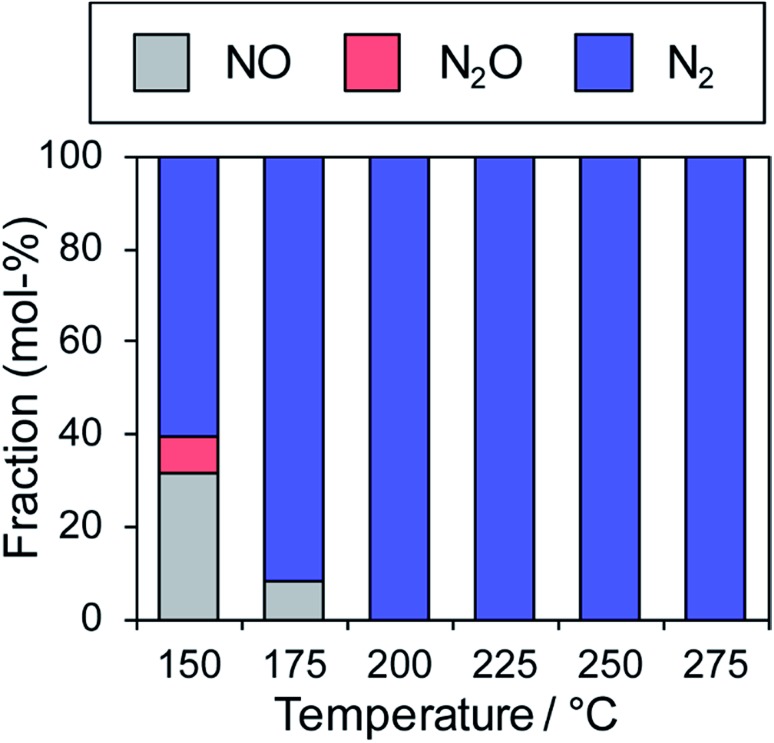
Fraction of NO, N_2_O and N_2_ in effluent gas (mol%) during catalytic NO reduction with CO using the Pd(In_0.33_Cu_0.67_)/Al_2_O_3_ catalyst (NO and CO: 0.5% balanced with He; catalyst: 0.060 g; total flow: 48 ml min^–1^).

In contrast, the Cu-dominant catalyst (*x* = 0.9) showed a typical light-off behavior and low N_2_ selectivity as observed for PdCu. Therefore, the characteristics of the parent bimetallic materials were strongly reflected in the catalysts with biased In/Cu ratios. The best performance was obtained when *x* = 0.67 with a monotonous increase in NO conversion and excellent N_2_ selectivity as observed for PdIn. It should be noted that the Pd(In_0.33_Cu_0.67_)/Al_2_O_3_ catalyst showed very high catalytic activities in low-temperature regions (*e.g.*, for NO conversion at 150 °C, Pd(In_0.33_Cu_0.67_) 42%, PdIn, 14%, and Pd 3%). Thus, the substitution of In with Cu remarkably enhanced the catalytic activity of PdIn without changing N_2_ selectivity. A control experiment using Cu/Al_2_O_3_ (Cu: 1.2 wt%) with the Cu content identical to that in Pd(In_0.33_Cu_0.67_)/Al_2_O_3_ was also performed because metallic Cu is known to be active for NO reduction by CO.^46^ However, under our conditions, Cu alone did not work at all below 275 °C, suggesting that the catalysis by Cu itself did not contribute to the significant enhancement in catalytic activity. We also tested the performance of the Pd(In_0.33_Cu_0.67_)/Al_2_O_3_ catalyst under various conditions. Fig. 6 shows the gas-phase distribution (mol%) of NO, N_2_O and N_2_ during catalytic NO reduction with CO using Pd(In_0.33_Cu_0.67_)/Al_2_O_3_ under standard conditions.

Emphasis should be placed on the complete conversion of NO into N_2_ at 200 °C and higher. To the best of our knowledge, this is the first report of complete deNO_*x*_ by CO at such a low temperature. N_2_O emission was not observed even at 175 °C, thus highlighting the remarkably high N_2_ selectivity. We also tested the long-term stability of the best catalytic performance at 200 °C. Although NO conversion was slightly decreased during the first several hours (100% to 72%), no further deactivation was observed up to 24 h (Fig. S5†). N_2_ selectivity was always 100%, showing that no N_2_O was emitted during the daylong operation. The catalytic performance was completely recovered (100% N_2_ yield, Fig. S5†) after the catalyst was placed in a reductive atmosphere (H_2_ flow, 400 °C for 0.5 h). Therefore, the initial deactivation occurs probably due to the accumulation of oxygen species on the catalyst surface, reaching a steady state during the early stages of the reaction. Besides, the Pd(In_0.33_Cu_0.67_)/Al_2_O_3_ catalyst exhibited excellent N_2_ selectivities (75–100%) even under a wide range of nonstoichiometric conditions (NO/CO ratio: 0.4–2.5; see ESI Fig. S6† for details). Note that high N_2_ selectivity was maintained under NO-rich (lean) conditions. The corresponding Pd/Al_2_O_3_ catalyst showed low N_2_ selectivities (27–61%) under these conditions, reflecting the difficulty of selective NO reduction using monometallic Pd catalysts. Thus, we developed a highly active and selective deNO_*x*_ catalyst using a Pd(In_0.33_Cu_0.67_) pseudo-binary alloy. The CO adsorption experiment confirmed that Pd(In_0.33_Cu_0.67_) had a higher Pd dispersion than PdIn (19 and 11%, respectively), as expected from the smaller particle size. This may be one of the reasons for the increased catalytic activity of Pd(In_0.33_Cu_0.67_). However, the great enhancement in NO conversion in the high-temperature region cannot be explained only by the improvement in metal dispersion. This point will be discussed later in greater detail. We also tested other trimetallic catalysts using various third metals (Pd(In_1–*x*_M_*x*_)/Al_2_O_3_, M = Fe, Co, Ni, and Ga). However, no catalytic performance superior to that of Pd(In_0.33_Cu_0.67_) was obtained (Fig. S7†).

### 3.3. Mechanistic study

**Fig. 7:**
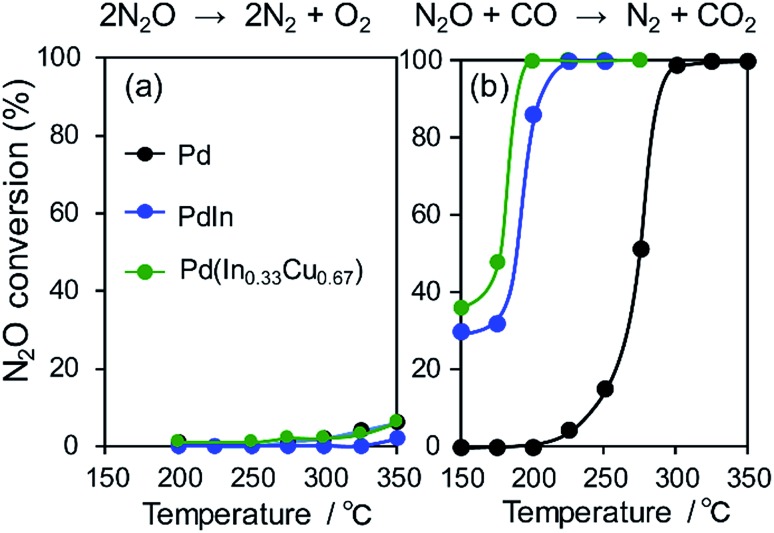
N_2_O (a) decomposition and (b) reduction by CO using Pd, PdIn, and Pd(In_0.33_Cu_0.67_) catalysts.

We then investigated the role of In and Cu in the great enhancement of N_2_ selectivity and NO conversion, respectively. As mentioned in the Introduction paragraph, N_2_ selectivity should be improved by inhibiting N_2_O formation and/or accelerating N_2_O decomposition. The latter can be easily evaluated using N_2_O as a reactant molecule. Therefore, we tested the catalytic activity of the Pd-based catalysts in N_2_O reduction to N_2_ with and without CO (Fig. 7).

Our results show that N_2_O conversion was very low (<5%) even at 350 °C in the absence of CO, whereas high N_2_ selectivity was obtained in NO reduction by CO. It has been known that N_2_O itself is easily decomposed to N_2_ and O on the clean surface of transition metals even at very low temperatures.^47^ However, a reaction no longer occurs after the saturation coverage of O on the surface. Therefore, this result indicates that (1) the catalyst surface is immediately covered with a small amount of O and (2) the formed surface oxide layers are hardly reduced without any reductant. In contrast, the N_2_O conversion trend was completely changed in the presence of CO. For Pd/Al_2_O_3_, N_2_O conversion was drastically increased as the temperature increased with a light-off temperature of 275 °C. PdIn/Al_2_O_3_ showed a similar trend but with a lower light-off temperature (175 °C). The light-off temperatures of N_2_O conversion were in good agreement with those of N_2_ selectivity in NO reduction by CO (Fig. 7b and 2b). These results strongly indicate that the remarkably enhanced N_2_O reduction ability contributes to the excellent N_2_ selectivity in NO reduction by CO.

**Fig. 8:**
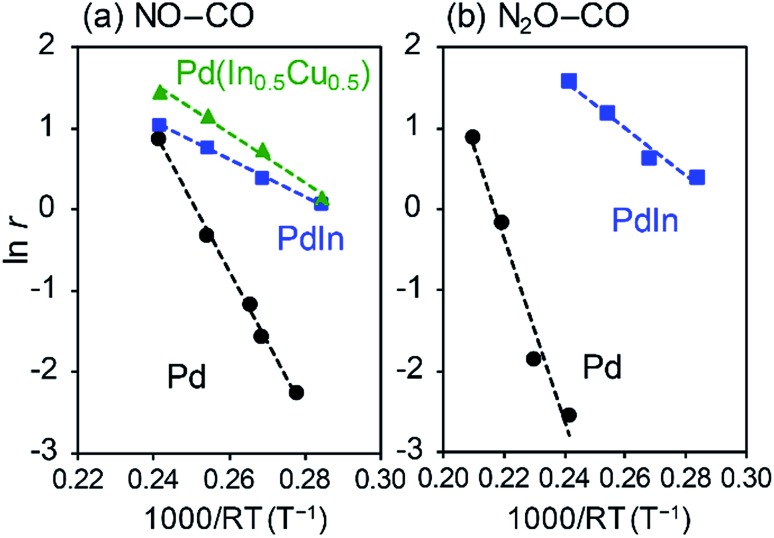
Arrhenius-type plots for (a) NO and (b) N_2_O reduction by CO using Pd/Al_2_O_3_, PdIn/Al_2_O_3_, and Pd(In_0.5_Cu_0.5_)/Al_2_O_3_ catalysts. Reaction rates (mol s^–1^ mol_Pd_^–1^) are evaluated based on the CO_2_ formation rate.

We then evaluated the apparent activation energies of N_2_O reduction by CO from Arrhenius-type plots (Fig. 8, Table 2 summarizes the results). Pd/Al_2_O_3_ and PdIn/Al_2_O_3_ showed activation energies of 113.4 and 28.9 kJ mol^–1^, respectively, indicating that the activation barrier was remarkably decreased by the formation of an intermetallic PdIn phase. A similar trend and *E*_A_ values were also observed in NO reduction by CO (86.5 and 23.1 kJ mol^–1^ for Pd/Al_2_O_3_ and PdIn/Al_2_O_3_, respectively). This implies that a common elementary step (*e.g.*, CO oxidation by surface O) limits the overall reaction rates in both N_2_O and NO reduction and is remarkably enhanced by PdIn. We also obtained the *E*_a_ from the Arrhenius plot based on the rate constant *k* (24.0 kJ mol^–1^ for PdIn/Al_2_O_3_, see ESI Fig. S8† for details), which was almost identical to that obtained based on the reaction rate *r*. Considering the significantly low activation energy, one might suspect that the reaction is diffusion-limited. However, we can exclude the possibility of diffusion-limited kinetics because PdIn shows catalytic activity similar to that of Pd (<30% conversion) under these conditions, where the reaction rate is limited obviously by some chemical reaction as represented by the high activation energy (86.5 kJ mol^–1^).

**Table 2:** Summary of Arrhenius-type plots and kinetic analysis

	N_2_O–CO		NO–CO		
	Pd	PdIn	Pd	PdIn	PdInCu^*a*^
*E*_A_/kJ mol^–1^	113.4	28.9	86.5	23.1 (24.0)^*b*^	30.8
Intercept	24.6	8.5	21.7	6.6 (7.6)^*b*^	8.9
Order for *P*_NO_	—	—	0.62	1.28	—
Order for *P*_CO_	—	—	0.55	0.08	—

RDS in NO–CO	*r* = *kP*_NO_^*x*^*P*_CO_^*y*^	
(1)	*x* = 1	–1 < *y* < 0
(2)	–1 < *x* < 1	*y* = 1
(3)	–1 < *x* < 3	–2 < *y* < 0
(4)	–6 < *x* < 0	–2 < *y* < 0
(5)	–4 < *x* < 2	–1 < *y* < 1
(6)	–2 < *x* < 0	–1 < *y* < 0
(7)	–2 < *x* < 0	–1 < *y* < 0
(8)	–1 < *x* < 1	–1 < *y* < 0

^*a*^Pd–In–Cu/Al_2_O_3_ (Pd : In : Cu = 2 : 1 : 1) was used as a catalyst.

^*b*^Estimated from the Arrhenius plot based on the rate constant *k*.

A similar low activation energy was also observed for Pd–In–Cu (30.8 kJ mol^–1^), suggesting that Cu-substitution did not strongly affect the kinetics in this temperature region. To understand the rate-determining step (RDS), a kinetic analysis was performed on the basis of the reaction orders for NO and CO pressures (*P*_NO_ and *P*_CO_) in NO reduction by CO (Fig. S9†). Table 2 summarizes the experimental reaction orders for *P*_NO_ and *P*_CO_, which were positive for both Pd and PdIn catalysts. The observed reaction orders (not equal to unity) also suggest that the reaction rate is not limited by gas diffusion. In this study, we considered an extended Langmuir–Hinshelwood mechanism with N_2_O decomposition/sorption processes as follows:

NO adsorption

1 NO + σ ⇌ NO·σ

CO adsorption

2 CO + σ ⇌ CO·σ

NO dissociation

3 NO·σ + σ ⇌ N·σ + O·σ

N_2_ formation

4 2N·σ → N_2_ + 2σ

CO oxidation (CO_2_ formation)

5 CO·σ + O·σ → CO_2_ + 2σ

N_2_O formation

6 N·σ + NO·σ ⇌ N_2_O·σ + σ

N_2_O decomposition

7 N_2_O·σ ⇌ N_2_ + O·σ

N_2_O desorption/readsorption

8 N_2_O·σ ⇌ N_2_O + σ

where σ indicates a vacant site. Steps (1)–(5) are identical to those considered in the conventional kinetic models for the NO–CO reaction over Pd^13^,^48^,^49^ and Rh^13^,^50^,^51^ catalysts.

The modified points are that N_2_O is once formed as an adsorbate (6) and that N_2_O decomposition and sorption are taken into account (7 and 8), which are the crucial factors for determining N_2_ selectivity in the present system. We solved the rate equation of each step regarded as the RDS using the overall site balance and equilibrium constants for other steps except (4) and (5), where reverse reactions can be ignored under atmospheric pressure conditions (see ESI† “Kinetic Analysis” for details). Table 2 shows the reaction orders for *P*_NO_ and *P*_CO_ derived from the equations. In most cases, the reaction order for *P*_CO_ is negative, which is inconsistent with the observed experimental result. Conversely, when (5) is considered rate-determining, the orders for *P*_NO_ and *P*_CO_ range from –4 to +2 and from –1 to +1, respectively, which agree with the experimental values. Thus, our kinetic study demonstrated that the oxidation of CO by surface oxygen was the RDS in NO reduction by CO. The order for *P*_NO_ was increased, whereas that for *P*_CO_ was decreased by the formation of the PdIn phase. This implies that NO adsorption becomes much less favorable than CO adsorption on PdIn. The observed features are quite different from those reported for conventional monometallic Pd catalysts, where the order for *P*_CO_ was typically negative^13^,^48^ and NO dissociation (3)^52^,^53^ or N_2_ formation (4)^49^ has been considered as the RDS. This discrepancy may stem from the difference in reaction temperature: our kinetic study was performed at a temperature (150 °C) that is much lower than that used in the literature (∼280 °C).^13^,^48^ CO oxidation can easily proceed at such high temperatures, where the RDS may be shifted to other steps. However, as clearly shown by the light-off temperature of CO conversion in the NO–CO reaction over Pd (Fig. S10†), CO oxidation seems to be very slow at temperatures lower than 200 °C. The enhancement of CO oxidation will provide a larger amount of vacant active Pd sites, thus allowing the promotion of NO and N_2_O adsorption and low-temperature activity and N_2_ selectivity, respectively. This effect will be discussed in detail later.

### 3.4. Operando XAFS

**Fig. 9:**
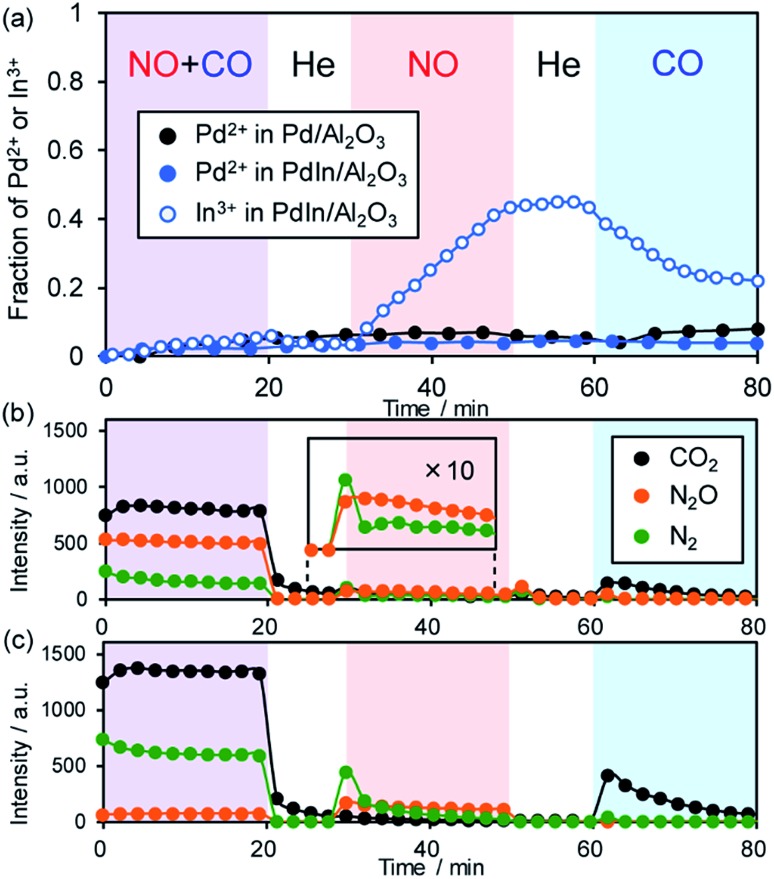
(a) Changes in the fraction of Pd^2+^ and In^3+^ species in Pd and PdIn catalysts during contact with NO + CO, NO, and CO at 200 °C. The corresponding changes in the product distribution in the gas phase for (b) Pd and (c) PdIn are also shown.

To clarify the role of the PdIn phase in the catalysis of NO reduction, we performed an operando XAFS study during reactions with NO and/or CO. The changes in the oxidation states of Pd and In were monitored on the basis of the features of XANES. Here we used 1 wt% Pd/Al_2_O_3_ and PdIn/Al_2_O_3_ catalysts, of which Pd dispersions are higher than those of 3 wt% catalysts (for PdIn, 18% and 11% for 1 wt% and 3 wt% catalysts, respectively), so that the information about the surface was included as much as possible in the overall information. After *in situ* H_2_ pretreatment at 400 °C, these catalysts showed Pd K (for Pd and PdIn) and In K (for PdIn) edge XANES spectra that are similar to those of zero-valent references (Pd black and In foil, respectively, Fig. S11a and b†), supporting the complete reduction of Pd and PdIn phases. For Pd K, the adsorption edge of PdIn was slightly lower in energy than that of Pd, suggesting that Pd atoms in PdIn are electron-enriched compared with those in pure Pd (Fig. S11a†); these findings are consistent with the result of CO-FT-IR. For In K edge XANES, the white line (1s → 5p transition) area of PdIn was larger than that of In foil (Fig. S11b†), indicating that the electron occupancy in the 5p orbital (In: [Kr] 5s^2^ 4d^10^ 5p^1^) decreased probably due to charge transfer from In to Pd. After the H_2_ pretreatment and a 10 min He purge, reactant gases (NO + CO mixture, NO, and CO) were flowed for 20 min with 10 min intervals of He purge (the changes in XANES spectra are shown in Fig. S11c and d†). Fig. 9a shows the change in the fraction of Pd^2+^ or In^3+^ species in the catalyst estimated by the linear combination fitting of the XANES spectra (see Fig. S12† for an example of the fitting).

The corresponding changes in the product distribution in the gas phase are shown in Fig. 9b and c. During contact with NO and CO, the fractions of the oxidized species were quite low (<0.05) for both Pd and PdIn catalysts (Fig. 9a). In the gas phase, N_2_, N_2_O, and CO_2_ were detected (Fig. 9b and c), showing that NO reduction by CO proceeded. The N_2_ selectivity was 22–32% and 89–92% for Pd and PdIn, respectively; these values are close to those observed in Fig. 2b. Thus, it was confirmed that the PdIn catalyst retained the metallic states of Pd and In during the catalytic NO reduction by CO. The presence of only a small amount of oxidized species indicates that oxygen atoms derived from NO dissociation are present at the catalyst surface but do not form any oxide phases. Note that their fractions are lower than the Pd dispersion, suggesting that the oxygen coverage does not reach saturation. In the case of PdIn, the fraction of In^3+^ was slightly higher than that of Pd^2+^. This implies that the surface oxygen atoms bind preferentially to In probably because of its intrinsic greater oxophilicity than Pd, *i.e.*, higher oxidation potential (In^(0)^ = In^3+^ + 3e^–^: 0.338 V; Pd^(0)^ = Pd^2+^ + 2e^–^: –0.915 V; *vs.* NHE).^54^

When only NO was introduced into Pd and PdIn catalysts, the fraction of In^3+^ in PdIn increased gradually with time up to *ca.* 0.4, whereas that of Pd^2+^ in Pd and PdIn did not change. Furthermore, small amounts of N_2_ and N_2_O were evolved in the gas phase during the initial stages of NO flowing, suggesting that NO dissociation and the subsequent N + N and N + NO reactions occur, but are inhibited rapidly by the accumulation of oxygen at the catalyst surface. For PdIn, considering the difference in In^3+^ and Pd^2+^ fractions, the oxygen atoms could also be captured by subsurface In to generate a Pd–In_2_O_3_ composite at the shell part of the PdIn nanoparticles. N_2_ was mainly evolved only at the earliest stage; thereafter, N_2_O formation dominated. This is probably because the association probability of two N atoms is decreased with increasing O coverage, thus making the N + NO reaction in an Eley–Rideal fashion favorable instead. Note that no significant difference in this trend was observed between Pd and PdIn, implying that In does not strongly inhibit the pathway of N_2_O formation.

Finally, when CO was introduced, the fraction of In^3+^ in the PdIn catalyst decreased with a small evolution of CO_2_. This clearly demonstrates that In_2_O_3_ was reduced by CO to form metallic In and CO_2_. However, the reduction of In_2_O_3_ was not completed under these conditions, suggesting the difficulty of removing oxygen from the bulk using CO as a reductant at 200 °C. In contrast, the fraction of Pd^2+^ in Pd and PdIn did not decrease during the contact with CO despite the formation of CO_2_ even on Pd. A possible interpretation is that the saturation coverage of CO on Pd sites and the resulting strong backdonation make Pd slightly electron deficient.

Thus, our operando XAFS analysis revealed that (a) In undergoes the redox cycle of In^3+^–In^(0)^*via* the reaction with NO and CO, (b) the parent PdIn phase is retained in the steady state of NO reduction by CO, and (c) In acts as an oxygen acceptor (in other words, an oxygen storage/release material). This redox property of PdIn can also be observed in our recent study on the oxidative dehydrogenation of 1-butene to 1,4-butadiene using O_2_ at 400 °C.^55^ In this case, intermetallic PdIn nanoparticles were oxidized by O_2_ to form a Pd–In_2_O_3_ composite, followed by the reconstruction of the parent PdIn nanoparticles by contact with 1-butene. The PdIn phase was retained in the copresence of O_2_ and 1-butene even at 400 °C. This self-reconstruction capability of PdIn would work even under three-way catalysis conditions, where reducing agents stronger than CO, such as hydrocarbons and hydrogen, are also present.

### 3.5. DFT calculations

**Fig. 10:**
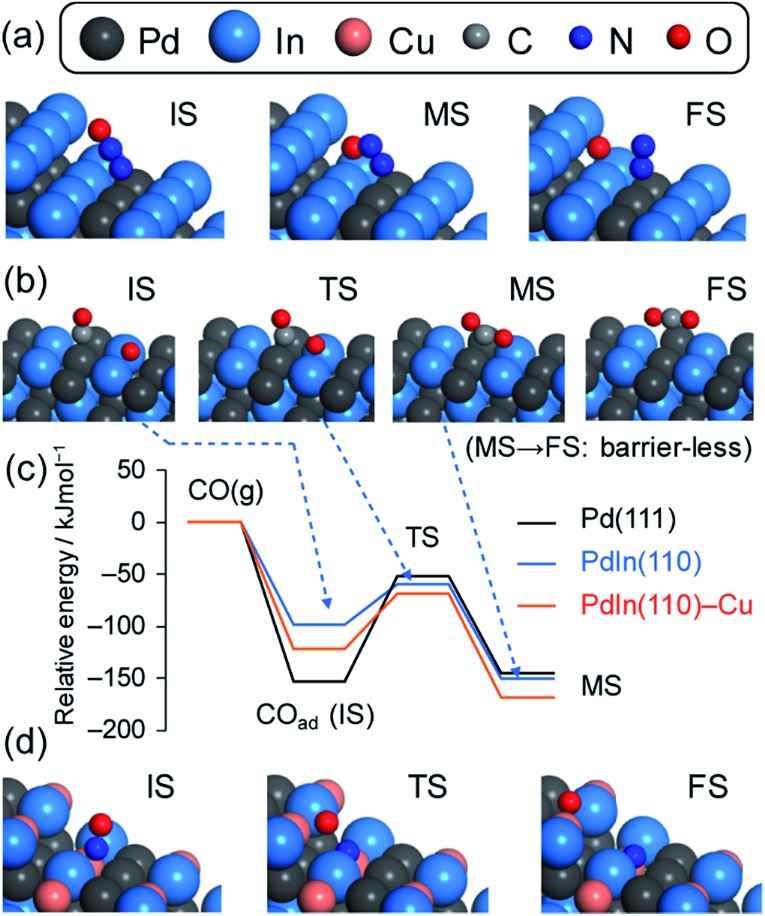
Molecular transformation calculated using DFT: (a) N_2_O decomposition over In-edged PdIn(120), (b) CO oxidation over PdIn(110), (c) energy diagram of CO oxidation, and (d) NO dissociation over Cu-substituted PdIn(120). IS, TS, MS, and FS indicate the initial, transition, intermediate, and final states, respectively. For (c), the total energy of the gas phase CO and O-preadsorbed slab was set to zero for each surface.

Finally, DFT calculations for the relevant elementary steps were performed to obtain further information on an atomic scale. To understand the selectivity trend, we focused on N_2_O decomposition (N_2_O → N_2_ + O), CO oxidation (O + CO → CO_2_), and N_2_ formation (N + N → N_2_) steps over the terrace and/or stepped surfaces of Pd and PdIn. The dissociation of NO (NO → N + O) was also studied in a similar fashion to follow the activity trend. Fig. 10 illustrates the views of representative adsorbates and Table 3 summarizes the adsorption energy (*E*_ad_), reaction energy (Δ*E*), and activation energy (*E*_A_) for each molecule and step (see ESI Fig. S13–S15† for the pictures of all species). For N_2_O decomposition, conversions from on-top linear N_2_O to bidentate bent N_2_O to dissociated N_2_ and O were considered.^56^ The second bond dissociation step typically gave higher *E*_A_ than the first molecular bending step except Pd(111), where the bending step showed a relatively high *E*_A_ (29.3 kJ mol^–1^).

**Table 3:** Adsorption (*E*_ad_), reaction (Δ*E*), and activation (*E*_A_) energies in various elementary steps over Pd and PdIn surfaces calculated using DFT

Surface	*E*_ad_/kJ mol^–1^	Δ*E*/kJ mol^–1^	*E*_A_/kJ mol^–1^	*E*_A_ reported	Ref.
**N**_**2**_ **O → N**_**2**_ **+ O** ^*a*^					
Pd(111)	–38.8	–110.2	9.3 (29.3)	(56.9)	52
Pd(100)	–53.8	–104.9	61.8 (13.5)		
Pd(511)	–54.6	–123.2	46.3		
PdIn(110)	–32.2	–107.7	18.6 (3.7)		
PdIn(120)	–60.0	–105.0	<0.1 (0)		

**O + CO → CO**_**2**_ ^*b*^					
Pd(111)	–153.0	–33.2	100.1	98.4	56
PdIn(110)	–98.9	–134.3	39.4		
PdIn(110)–Cu	–122.0	–44.6	53.4		

**N + N → N**_**2**_					
Pd(111)		–190.2	77.9		
PdIn(110)		–353.4	86.1		

**NO → N + O**					
Pd(100)	–276.0	158.1	158.1	157.3	58
Pd(511)	–257.0	41.5	100.2	115.8	58
PdIn(110)	–110.3	46.8	167.3		
PdIn(120)	–120.9	27.2	111.8		
PdIn(120)–Cu	–191.6	–7.0	100.2		

^*a*^Energies for bidentate N_2_O are shown. *E*_A_ for the bending step (from linear to bidentate N_2_O) is shown in parenthesis.

^*b*^ *E*_ad_ for CO adsorption on the O-preadsorbed (not clean) surface is shown.

The PdIn(110) terrace gave an *E*_A_ that is significantly lower than those of all Pd surfaces calculated. It is surprising that N_2_O decomposition on In-edged PdIn(120), which is stepped (110), was almost barrierless (*E*_A_ < 0.1 kJ mol^–1^). Therefore, PdIn surfaces are much more active for N_2_O decomposition than Pd. This may be due to the oxophilic character of metallic In, which is likely to provide significantly stabilized TSs for bond dissociation. In contrast, the *E*_ad_ of N_2_O was close to each other between the PdIn and Pd surfaces.

CO oxidation over the PGM surface has been extensively studied using surface science techniques^57^–^59^ and theoretical calculations.^60^,^61^ For example, the calculated activation energies for Pd(111) range from 90 to 144 kJ mol^–1^ depending on the coverage.^60^,^61^ Our calculation for 1/4 of the coverage of CO on Pd(111) (100.1 kJ mol^–1^) well reproduced the corresponding reported value (98.4 kJ mol^–1^)^60^ and it was close to the experimental value obtained in the present study (113 kJ mol^–1^). In contrast, CO oxidation over PdIn(110) gave a much lower *E*_A_ (39.4 kJ mol^–1^) than Pd(111) and was close to that obtained from the Arrhenius-type plot (29 kJ mol^–1^). Thus, our calculation showed good agreement with the reported theoretical and the present experimental values. The remarkable decrease in *E*_A_ for PdIn could be explained by the weaker adsorption of CO (less negative *E*_ad_: –98.9 > –153.0 kJ mol^–1^). This is clearly represented in the energy diagram for CO adsorption and the subsequent oxidation (Fig. 10c): the relative total energies are similar, except for the adsorbed state of CO (initial state of CO oxidation). The weaker adsorption of CO makes the initial state unstable, resulting in a lower energy barrier. We also considered the effect of Cu substitution in PdIn on the CO oxidation ability. The substitution of some In atoms with Cu atoms at the PdIn(110) surface resulted in a more negative *E*_ad_ and a slightly higher *E*_A_, indicating that Cu substitution does not enhance the CO oxidation ability of PdIn.

Considering the large difference between the *E*_A_ of N_2_O decomposition and CO oxidation, CO oxidation should be the RDS for the N_2_O–CO reaction. Therefore, the remarkable decrease in the activation energy of CO oxidation can be the reason why the N_2_O–CO activity and N_2_ selectivity in the NO–CO reaction were drastically improved. Our calculations also revealed that N_2_O decomposition itself was also greatly promoted over the PdIn surface. This promotion effect may also contribute to the overall N_2_ selectivity in the NO–CO reaction. We also focused on the N_2_ formation step because the acceleration of this step can contribute to enhanced N_2_ selectivity in the NO–CO reaction. However, no significant difference in *E*_A_ was observed between Pd(111) and PdIn(110) surfaces.

NO dissociation over transition metal surfaces has also been long studied for CO oxidation.^27^,^62^–^65^ Sautet *et al.*^27^ reported that the *E*_A_ of NO dissociation at the step of Pd(511) was 115.8 kJ mol^–1^, which was much lower than that on Pd(111) and (100) terraces (216 and 160 kJ mol^–1^, respectively). Our DFT calculation for monometallic Pd surfaces reproduced the reported *E*_A_ (100.2 and 158.1 kJ mol^–1^ for (511) and (100) surfaces, respectively). A similar trend in *E*_A_ was observed for PdIn, where the (120) surface (stepped (110)) showed a much lower *E*_A_ than the flat (110) surface (111.8 and 167.3 kJ mol^–1^, respectively). The decrease in *E*_A_ at the step sites can be rationalized by a geometric effect. Given that the O atom of NO in the on-top conformation is distant from the surface, NO dissociation at the terrace sites requires large atomic displacement without stabilization. Conversely, the step sites allow lower atomic displacement and stabilization of TSs owing to the spatial adjacency of the metal atoms and O (Fig. S15c–e†). These suggest that NO dissociation over PdIn, as well as that over pure Pd and Rh frequently advocated in the literature, is structure sensitive.^27^,^49^,^62^,^66^ However, the *E*_A_ of PdIn surfaces was slightly higher than that of Pd surfaces. Moreover, *E*_ad_ on PdIn was much less negative than that of Pd as observed for CO. The weakening of NO adsorption on PdIn was more prominent than that of CO adsorption (*E*_ad_ of NO: –276.0 → –110.3 kJ mol^–1^, *E*_ad_ of CO: –153.0 → –98.9 kJ mol^–1^). This result indicates that CO adsorption on PdIn is relatively favored compared with that on Pd, consistent with the experimental change in the reaction orders of *P*_NO_ and *P*_CO_ mentioned in the kinetic study. Therefore, the PdIn surface has no promotion effect on NO adsorption and dissociation compared with pure Pd. By contrast, the Cu-substituted PdIn(120) surface, which is a model of a Pd(In_1–*x*_Cu_*x*_) pseudo-binary alloy surface (Fig. 10d), showed a different trend. The *E*_ad_ of NO becomes more negative (–120.9 → –191.6 kJ mol^–1^) by Cu substitution. *E*_A_ was also decreased to the level of Pd(511). Thus, Cu substitution enhanced both NO adsorption and dissociation, consistent with the improved catalytic activity in the NO–CO reaction. The decrease in *E*_A_ by Cu-substitution should be attributed to the drastically lowered Δ*E* from positive to negative (+27.2 → –7.0 kJ mol^–1^). This result could be attributed to the fact that the final dissociated state is significantly stabilized by the incorporation of Cu, which has greater azophilicity (affinity to N) than typical metal elements.^67^ Thus, Cu substitution from In drastically modified the electronic feature of the catalyst and the catalytic performance.

Finally, we investigated the effect of the electronic state on the observed catalysis. We focused on the characteristics of valence electrons, which strongly affect the adsorptivity and reactivity. It is known that the d-band center can be a good descriptor for these surface chemical behaviors.^68^–^71^ Generally, the lower the center is, the weaker the adsorption is. We calculated the density of states (DOS) diagrams projected onto the d orbitals of the relevant catalyst surfaces: Pd(111), PdIn(110), PdCu(110), and Pd(In_0.33_Cu_0.67_)(110) (Fig. S16a†). The order of the d-band center position was PdCu (–2.07 eV) > Pd (–2.32 eV) > Pd(In_0.33_Cu_0.67_) (–2.64 eV) > PdIn (–3.05 eV). The d-band of PdIn was significantly down-shifted below –2.0 eV, because of the hybridization with low energy 4d electrons of In (<–10 eV).^72^ On the other hand, PdCu has an intense DOS peak near –1.1 eV derived from Cu,^73^ which uplifted the d-band center. Pd(In_0.33_Cu_0.67_) displayed an intermediate DOS diagram due to the contribution of both In and Cu, thus showing a moderate d-band center. The order of the d-band center agreed finely with those of *E*_ad_ of NO and CO and *E*_A_ of CO oxidation (Fig. S16b†): strong positive correlations were observed between the d-band center and the adsorption strength or the energy barrier. Therefore, the observed trend in the electronic states and chemical behaviors could be understood based on the d-band theory.

## 4. Discussion

On the basis of the obtained results, we summarize the outline of NO reduction by CO over PdIn and Pd(In_0.33_Cu_0.67_) catalysts and clarify the role of In and Cu in the great enhancement of the catalytic performances with respect to selectivity and catalytic activity.

### 4.1. Effect on N_2_ selectivity

CO oxidation is very slow in the low-temperature region (<225 °C), thus limiting the overall reaction rate of NO and N_2_O reduction. The coverages of CO and O are likely to be high, which strongly inhibits re-adsorption of N_2_O and its subsequent decomposition to N_2_ and O. Considering the sufficiently low *E*_A_ of N_2_O decomposition over Pd and PdIn, the decomposition should smoothly proceed once N_2_O is adsorbed with the bidentate conformation. Therefore, providing vacant active Pd sites is an important factor for N_2_O decomposition, particularly at low temperatures. PdIn promotes CO oxidation, thus providing more active sites.

The other key factor for N_2_ selectivity can be the difference in N_2_O capturing and decomposing abilities. Considering the large difference in the *E*_ad_ of N_2_O and NO or CO (*ca.* 220 kJ mol^–1^ for Pd(100)), N_2_O adsorption on Pd is extremely inhibited by the strong competitive adsorption of NO and CO. Therefore, N_2_O adsorption and decomposition may not be sufficiently enhanced only by releasing active sites. In contrast, the adsorption of NO and CO on PdIn is drastically weakened as demonstrated by the kinetic and DFT studies, whereas the *E*_ad_ of N_2_O was almost the same between Pd and PdIn. It should be noted that the difference in the *E*_ad_ of N_2_O and NO is decreased to *ca.* 60 kJ mol^–1^ on PdIn(120). This result indicates that N_2_O adsorption on PdIn in the presence of NO and CO is more favorable than that on Pd. This is probably because oxophilic In captures the oxygen atom of N_2_O in the bidentate conformation. Besides, PdIn possesses a greater ability to decompose N_2_O than Pd, as highlighted in the barrierless conversion at the stepped surface. Considering that N_2_O desorption competes with N_2_O decomposition, the faster N_2_O decomposition should play an important role in the overall N_2_ selectivity. Thus, the PdIn surface provides more vacant active sites, captures N_2_O more favorably, and decomposes it very smoothly. We concluded that the triple combination of these effects for N_2_O decomposition allows excellent N_2_ selectivity in NO reduction by CO even at low temperatures.

### 4.2. Effect on low-temperature activity

CO oxidation is the RDS of NO reduction by CO in the low temperature region (<225 °C). The PdIn phase significantly accelerates this step owing to the lowered activation barrier, resulting in the enhancement of the overall reaction rate. In other words, NO conversion is increased by releasing a larger amount of vacant active sites. In this context, it is interesting that the promotion of CO oxidation contributes to the enhancement of both selectivity and activity. We also propose another effect of PdIn on the low-temperature activity. As revealed by the DFT calculation and kinetic study, CO adsorption on PdIn is relatively favored compared with NO adsorption. Indeed, considering that the reaction order of *P*_CO_ is close to zero, the CO coverage seems to reach near saturation. Therefore, the higher concentration of adsorbed CO should increase the reaction rate of CO oxidation. Thus, CO oxidation over PdIn is accelerated by decreased *E*_A_ and increased CO coverage. By contrast, as mentioned in the paragraph of DFT calculations, the substitution of In with Cu does not enhance CO oxidation, thus causing low-temperature activity in NO reduction. Therefore, the observed increase in NO conversion by Cu-substitution in the low temperature region may be attributed to the improved metal dispersion.

Here, one may wonder why CO oxidation on PdIn becomes the RDS even though the corresponding *E*_A_ (∼30 kJ mol^–1^) is much lower than that of other steps such as NO dissociation (*E*_A_: ∼100 kJ mol^–1^). Considering the large difference in *E*_A_ between Pd and PdIn (∼70 kJ mol^–1^), the rate constants are expected to differ on the order of 10^7^ at 200 °C, which is much higher than the actual increase in the reaction rate on the order of 10^1^. This discrepancy can be explained by the difference in the pre-exponential factors. As suggested in the Arrhenius-type plots (Fig. 8), the intercepts of the linear lines significantly differ (Pd: 21.7, PdIn: 6.6, Table 2), which roughly shows that the pre-exponential factors differ by several orders of magnitude. In the literature of CO oxidation, such small pre-exponential factors have been observed^74^ and are accompanied typically by low apparent activation energies and high coverages. This is probably because of the rarity of CO oxidation on PdIn, in which the reaction pathway is geometrically limited by highly ordered atomic arrangement and the specific location of CO and O (Pd top and PdIn_2_ hollow sites, respectively, Fig. 10b and S14d†).

### 4.3. Effect on high-temperature activity

In contrast to the low-temperature region, the kinetics of NO reduction by CO changes drastically in a high-temperature region (>225 °C). CO oxidation smoothly proceeds (Fig. S10†), and hence it is no longer the RDS. NO dissociation, which has significantly high *E*_A_, is more likely to be rate-determining. For PdIn, the lower NO adsorption and dissociation abilities decrease the overall reaction rate, which is reflected in the low NO conversion in the high temperature region (Fig. 2a). This drawback of PdIn can be improved by the partial substitution of In with Cu as shown in Fig. 5. The high-temperature activity (NO conversions at 250 and 275 °C) increased as the Cu content in Pd(In_1–*x*_Cu_*x*_) increased. This is because NO adsorption and dissociation are facilitated by the incorporation of Cu, as demonstrated by the DFT calculation. However, the excess amount of Cu (*x* = 0.9) results in a sharp drop in the low-temperature activity (NO conversions at 150–200 °C), which is probably due to excessively strengthened CO adsorption. The low- and high-temperature activities derived from In and Cu, respectively, are compatible at the optimum Cu content (*x* = 0.67), thus affording remarkably high catalytic activity and selectivity in a wide range of reaction temperatures.

### 4.4. Effect of electronic factor

As summarized in Fig. S16,† In significantly lowers the d-band center, while Cu recovers the drop close to the level of Pd. The lowered d-band center makes NO and CO adsorption weaker. The weaker CO adsorption decreases *E*_A_ for CO oxidation because the TS energies are almost identical regardless of the surface (Fig. 10c). This effect contributes to the enhanced catalytic activity in the low temperature region. On the other hand, the weaker NO adsorption inhibits NO dissociation, which suppresses the catalytic activity in the high temperature region. NO adsorption is promoted with the aid of Cu substitution, which contributes to the greater catalytic activity in the high temperature region. However, the trend in NO dissociation ability is not straightforward, since other factors such as the intrinsic azophilicity of Cu, Δ*E*, and/or the adsorption configuration are also involved.

## 5. Conclusion

In the present study, we prepared a series of Pd-based intermetallic compounds supported on alumina (PdM/Al_2_O_3_, M = Cu, In, Pb, Sn, and Zn) and tested them in NO reduction by CO. PdIn exhibited a remarkably high N_2_ selectivity in a wide range of temperatures (>200 °C) and high NO conversion compared to pure Pd in a low-temperature region (<200 °C), whereas low conversion was observed in the high-temperature region (>200 °C). The catalytic performance of PdIn was remarkably improved by the substitution of a part of In with Cu to form Pd(In_1–*x*_Cu_*x*_) pseudo-binary alloys, achieving high NO conversion and N_2_ selectivity in various temperature regions (>175 °C). The optimized catalyst, namely, Pd(In_0.33_Cu_0.67_)/Al_2_O_3_, allows the complete conversion of NO to N_2_ even at 200 °C, which has never been achieved using metallic catalysts to the best of our knowledge. The formation of the pseudo-binary alloy structure with In–Cu substitution was supported by HAADF-STEM and EXAFS analyses, where the lattice constant was decreased by the incorporation of Cu atoms smaller than In atoms and no In–Cu bond was observed in the first coordination shell. The mechanistic study based on kinetic analysis and DFT calculation revealed the role of In and Cu as follows: (1) In accelerates CO oxidation, which is the RDS in the low-temperature region, thus providing a higher number of vacant active sites for NO dissociation (low-temperature activity) and N_2_O decomposition (N_2_ selectivity); (2) N_2_O decomposition ability itself is also enhanced by the formation of the PdIn phase; and (3) the incorporation of Cu improves NO adsorption and dissociation, which may be the RDS in the high-temperature region. Thus, the combination of the positive effects of In and Cu enables the development of highly active and selective NO reduction by CO in various temperature regions. Note that this combination is allowed only when In atoms in the PdIn phase are replaced by Cu with an appropriate ratio. The present study provides not only a highly efficient catalytic system for deNO_*x*_, but also deep insights into NO reduction and CO oxidation and a novel concept for flexible catalyst design based on the pseudo-binary alloy structure.

## Conflicts of interest

There are no conflicts to declare.

## Supplementary Material

Supplementary information
