# Supplementary material for: Design of Pd-based pseudo-binary alloy catalysts for highly active and selective NO reduction
Source: Chem Sci. 2019 Mar 4;10(15):4148–62. doi: 10.1039/c8sc05496g (PMC6471737; doi:10.1039/c8sc05496g)
Supplement: Supplementary file 1 [file SC-010-C8SC05496G-s001.pdf]

Electronic Supplementary Material (ESI) for Chemical Science.
This journal is © The Royal Society of Chemistry 2019

Supporting Information

Design of Pd-Based Intermetallic Catalysts for Highly Active and Selective NO Reduction

Jaewan Jeon,[†] Ken-ichi Kon,[†] Takashi Toyao,[†,‡] Ken-ichi Shimizu[†,‡],

Shinya Furukawa,*[,†,‡]

[†]*Institute for Catalysis, Hokkaido University, N-21, W-10, Sapporo 001-0021, Japan*

[‡]*Elements Strategy Initiative for Catalysts and Batteries, Kyoto University, Katsura, Kyoto 615-8520, Japan*

E-mail: furukawa@cat.hokudai.ac.jp,

Tel: +81-11-706-9162, Fax: +81-11-706-9163

Table S1. Summary of nanoparticle property for Pd-based catalyst supported on Al_2O_3.

property	Pd	PdIn	Pd($In_{0.33}Cu_{0.67}$)
d_{XRD} / nm [a]	7.3	6.6	– [b]
d_{STEM} /nm [c]	–	7.2	3.3
Pd dispersion (%)	13.6	11.1	19.4
$d_{(110)}$ / Å [d]	–	2.30	2.11
space group	$Fm\bar{3}m$	$Pm\bar{3}m$	$Pm\bar{3}m$

[a] Crystallite size estimated applying Sherrer equation for the most intense diffraction peak. [b] The diffraction peak was too broad to apply sherrer equation. [c] Volume weighted average size of the nanoparticles evaluated from STEM observation. [d] Interplanar distance of (110) planes of the $Pm\bar{3}m$ crystals estimated from STEM observation.

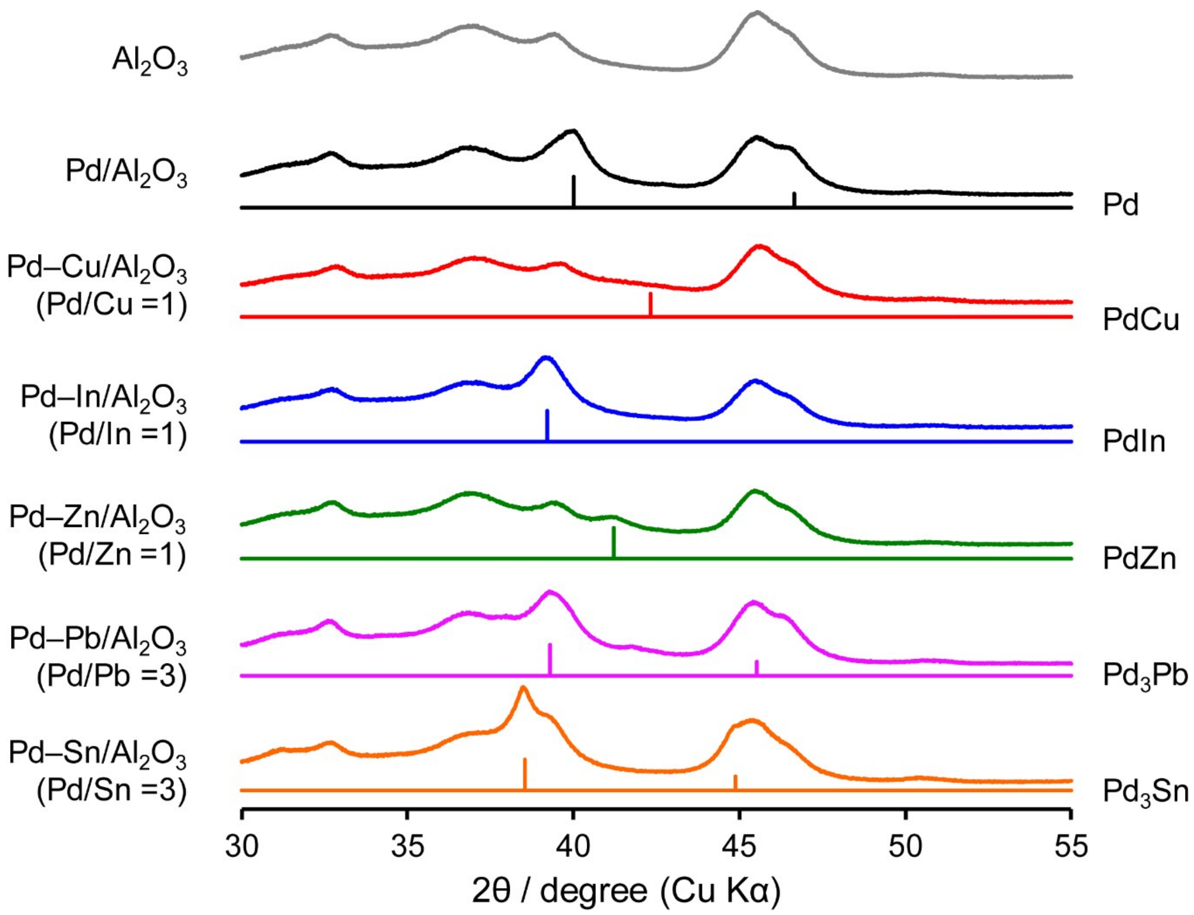

Figure S1. XRD patterns of alumina-supported Pd-based bimetallic catalysts. The standard diffraction pattern is shown below each pattern.

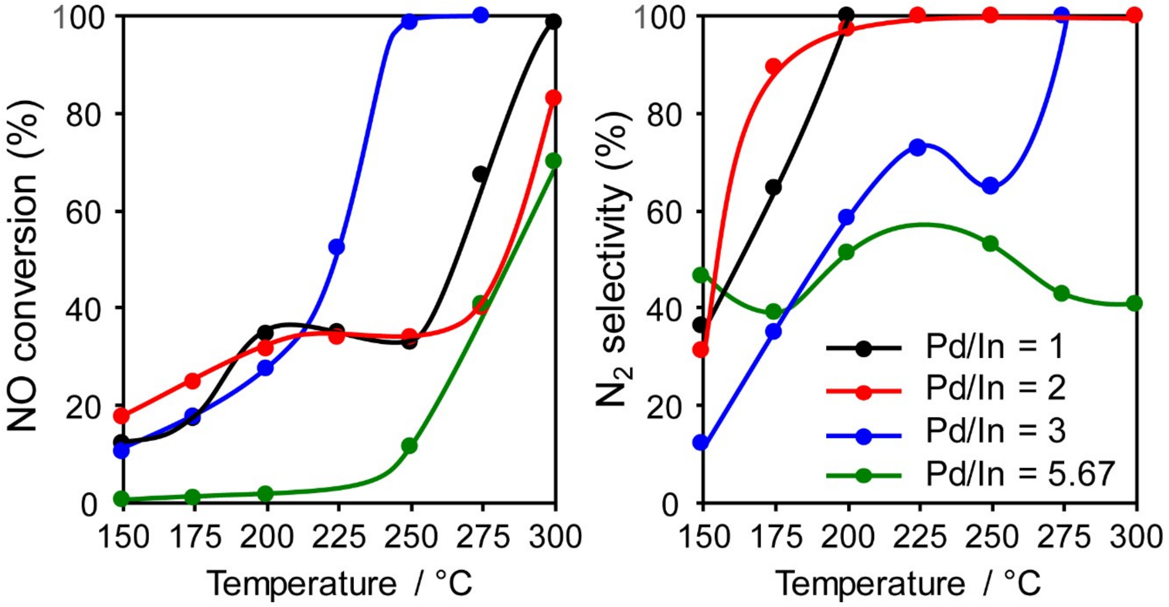

Figure S2. NO conversion and N_2 selectivity in NO reduction by CO using Pd–In/Al_2O_3 catalyst with various Pd/In ratios.

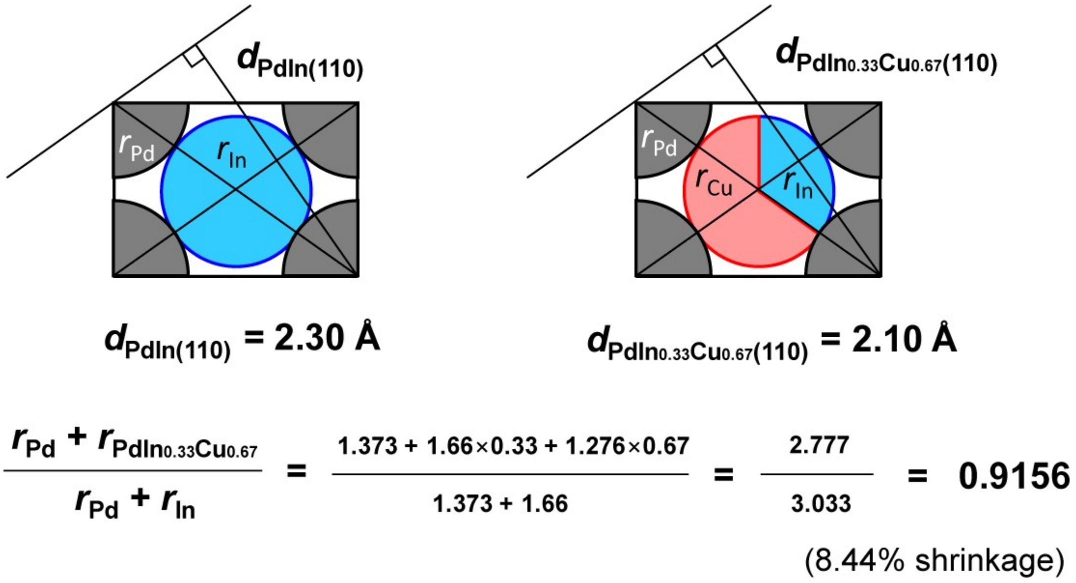

Figure S3. Theoretical shrinkage of PdIn lattice applying Vegard's law with each atomic size and ratio (Pd: 1.373 Å, In: 1.660 Å, Cu: 1.276 Å, 3:1:2) at (110) plane.

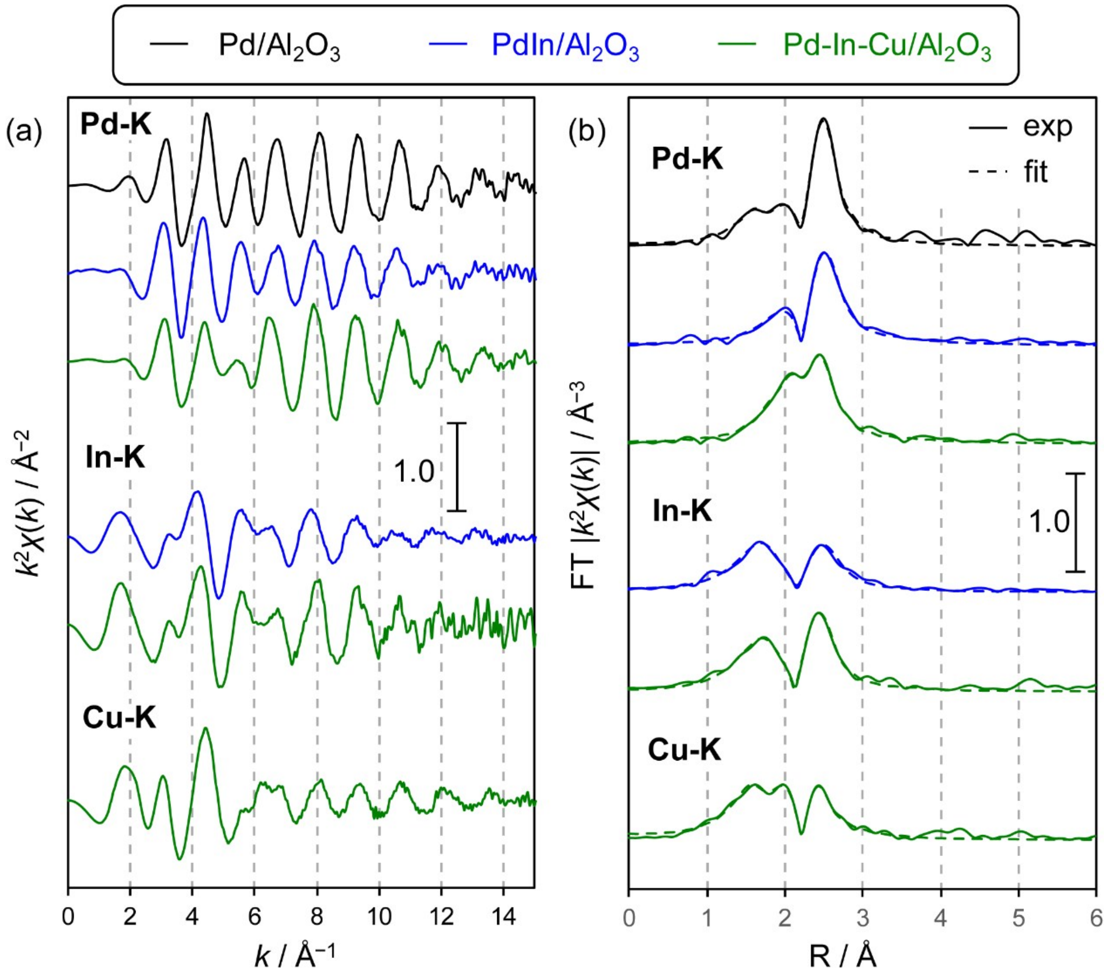

Figure S4. (a) k^2-weighted EXAFS oscillations of Pd/Al_2O_3, PdIn/Al_2O_3, and Pd-In-Cu/Al_2O_3 (Pd:In:Cu = 3:1:2) and their (b) Fourier-transforms (solid lines) and curve fits (dashed lines).

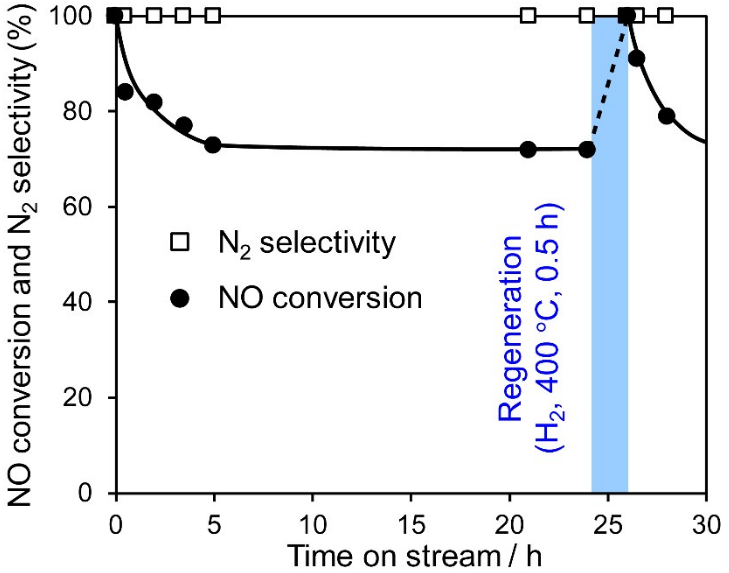

Figure S5. Chanes in NO conversion and N_2 selectivity during a long time NO−CO reaction over Pd($In_{0.33}Cu_{0.67}$)/Al_2O_3 (NO and CO: 0.5% balanced with He; catalyst: 0.060 g; total flow: 48 mlmin^{-1}).

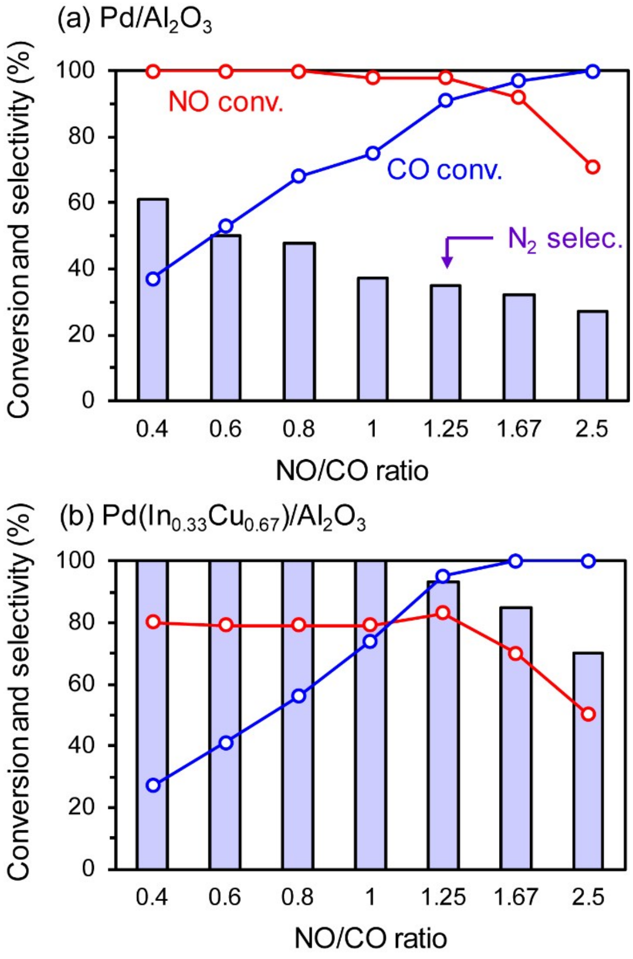

Figure S6. Catalytic performances of (a) Pd/Al_2O_3 and (b) $Pd(In_{0.33}Cu_{0.67})/Al_2O_3$ catalysts in NO reduction by CO with various NO/CO ratio.

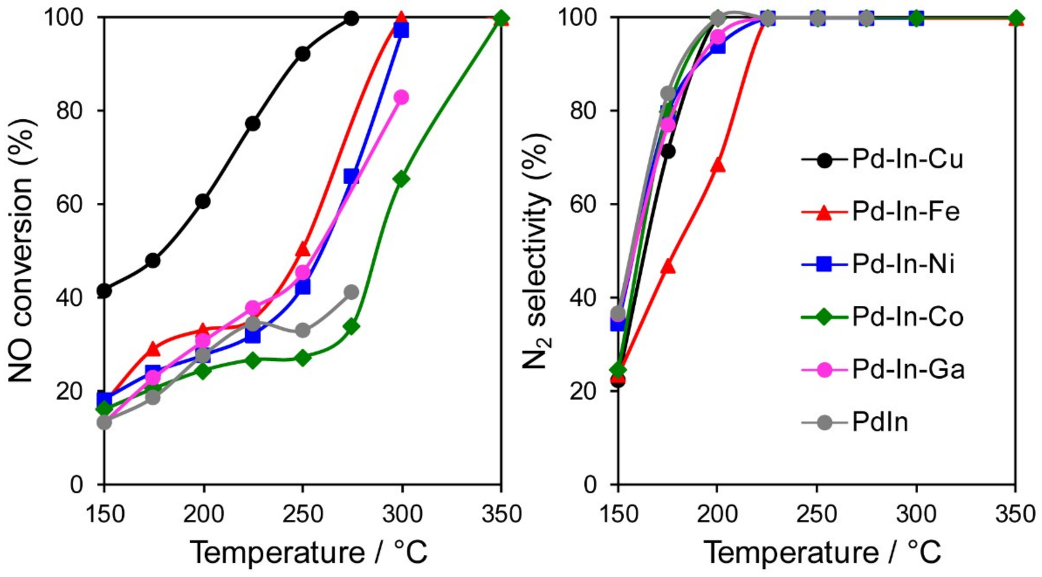

Figure S7. Catalytic performances of $Pd(In_{0.33}M_{0.67})/Al_2O_3$ catalysts in NO reduction by CO.

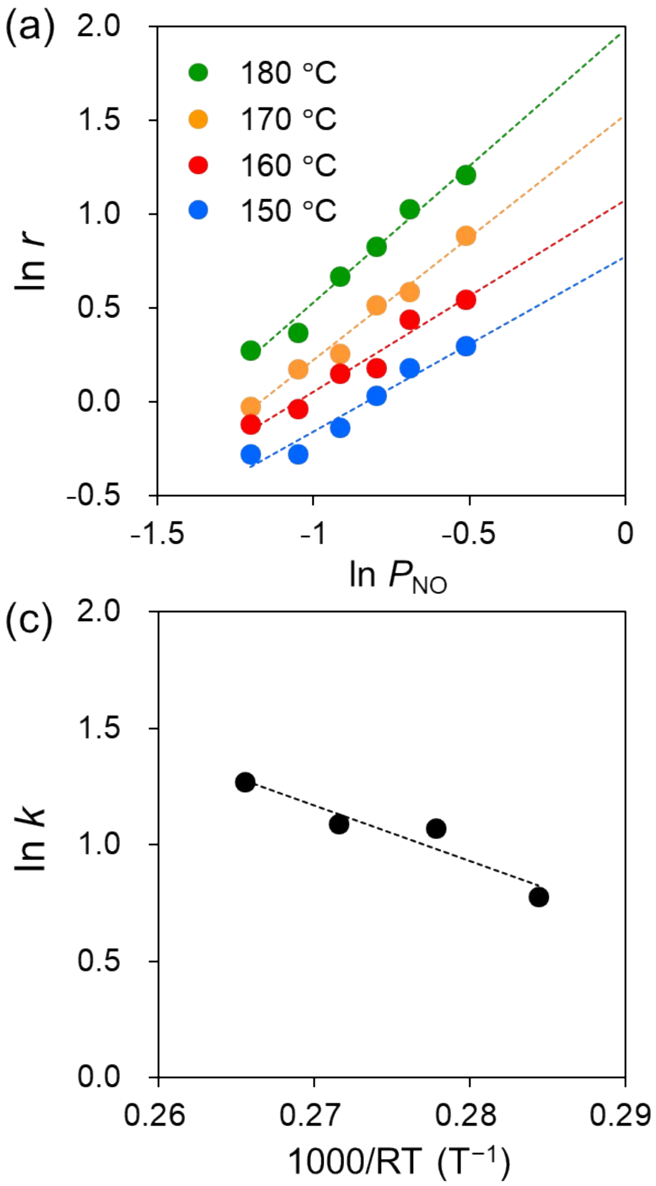

(b)

$$r = kP_{NO}{}^{\alpha}P_{CO}{}^{\beta}$$

$$\ln r = \ln k + \alpha \ln P_{NO} + \beta \ln P_{CO}$$

$$\ln r = \ln k + \alpha \ln P_{NO} \quad (\beta \approx 0)$$

$$\ln k = \ln A - \frac{E_a}{RT}$$

(d) summary of (a)

temp. / °C	slope (α)	intercept ($\ln k$)
150	0.93	0.78
160	1.02	1.07
170	1.30	1.09
180	1.45	1.27

summary of (c)

slope ($-E_a$)	intercept ($\ln A$)
−24.0	7.6

Figure S8. (a) Dependence of reaction rate on NO partial pressures (P_{NO}) in NO reduction by CO over PdIn/Al_2O_3. (b) Rate equations for NO reduction by CO. β can be regarded as zero from the result of Figure S9. This approximation allows to obtain ln k as the intercept of the linear line in (a). (c) Arrhenius plot for NO reduction by CO over PdIn/Al_2O_3. (d) Summary of the data from (a) and (c). Note that the slope α ranges from 0.9 to 1.5, which is consistent with the reaction order suggested from the kinetic analysis in Table 2.

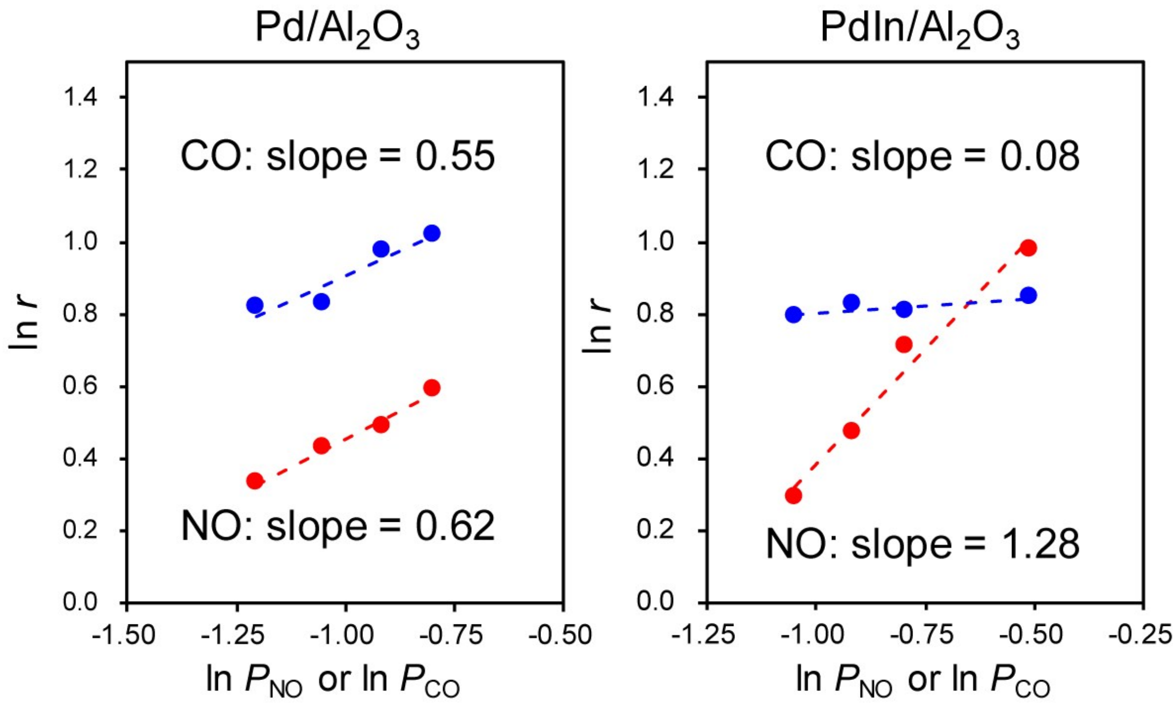

Figure S9. Dependence of reaction rate on NO and CO partial pressures (P_{NO} and P_{CO}, respectively) in NO reduction by CO over Pd/Al_2O_3 and $PdIn/Al_2O_3$.

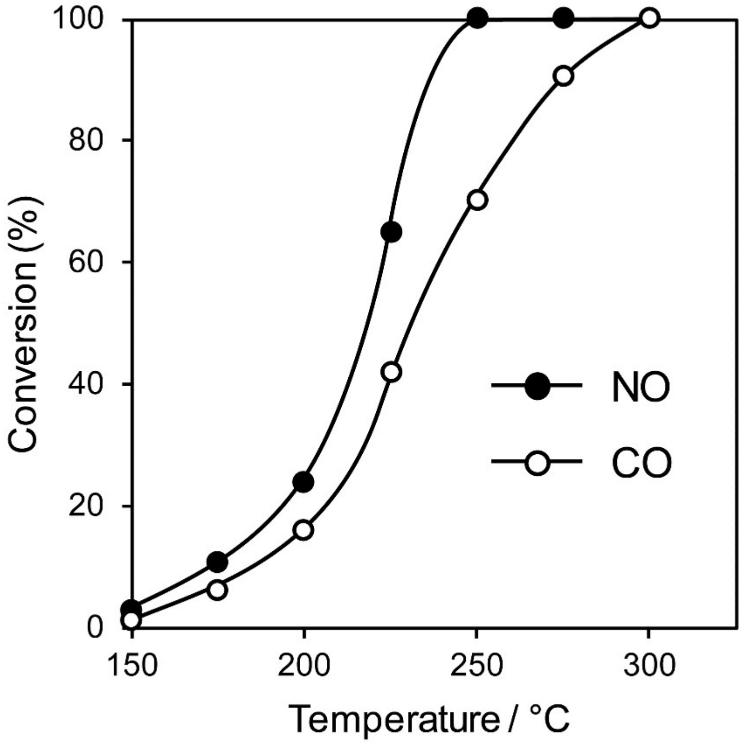

Figure S10. Conversion of NO and CO in NO reduction by CO over Pd/Al_2O_3 catalyst.

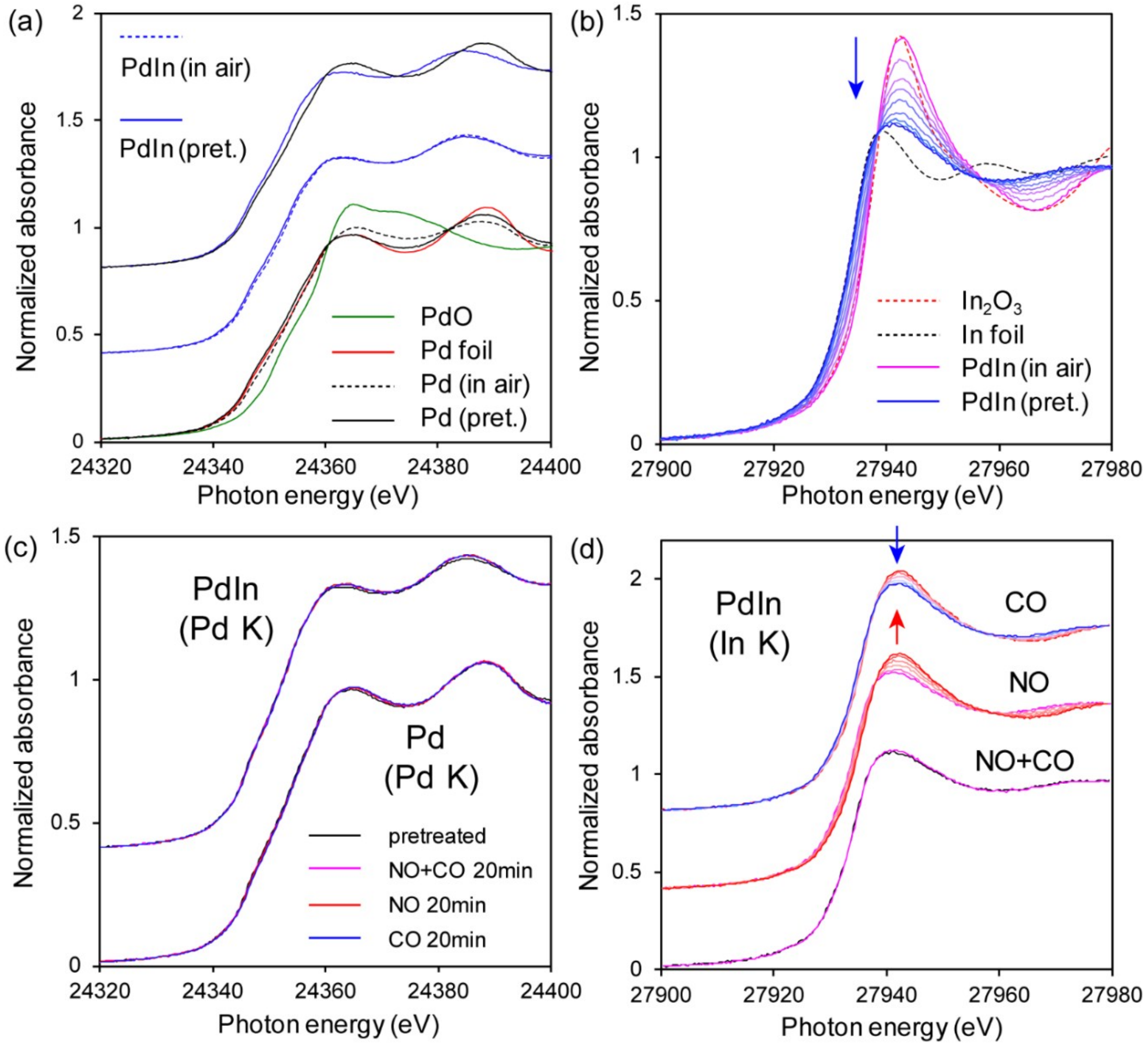

Figure S11. (a) Pd K and (b) In K-edge XANES spectra of Pd/Al_2O_3 and PdIn/Al_2O_3 catalyst before (in air) and after (pret.) H_2 pretreatment at 400°C for 20 min. Changes in the XANES spectra (c) after and (d) during 20 min contact with flowing NO+CO, NO, and then CO at 200°C.

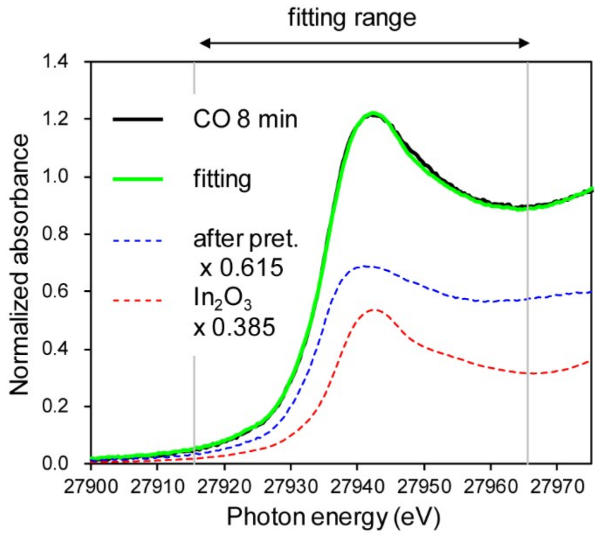

Figure S12. Linear combination fitting for a XANES spectrum during NO reduction by CO.

(a) N_2O decomposition over Pd(111)

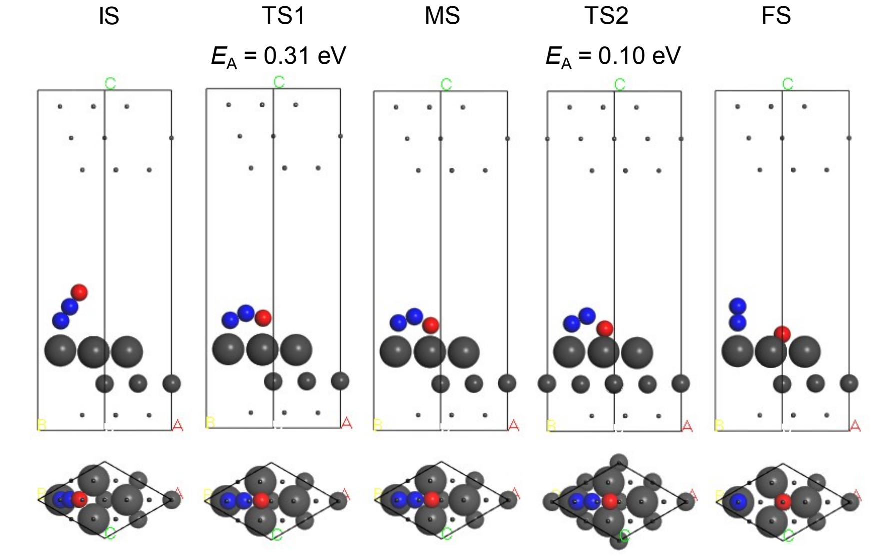

(b) N_2O decomposition over Pd(100)

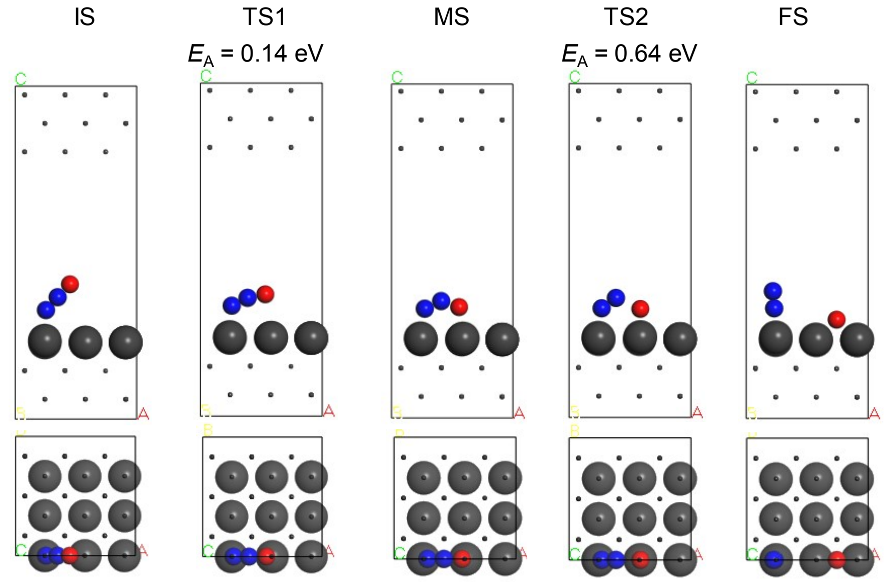

(c) N_2O decomposition over Pd(511)

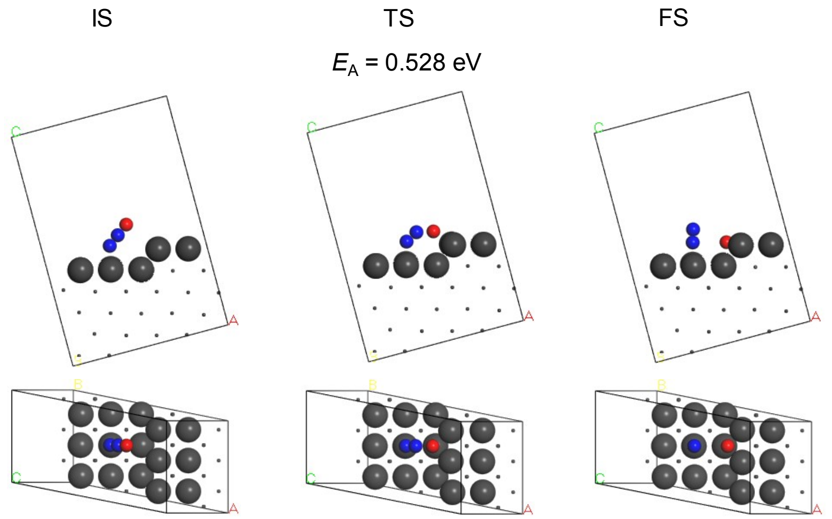

(d) N_2O decomposition over PdIn(110)

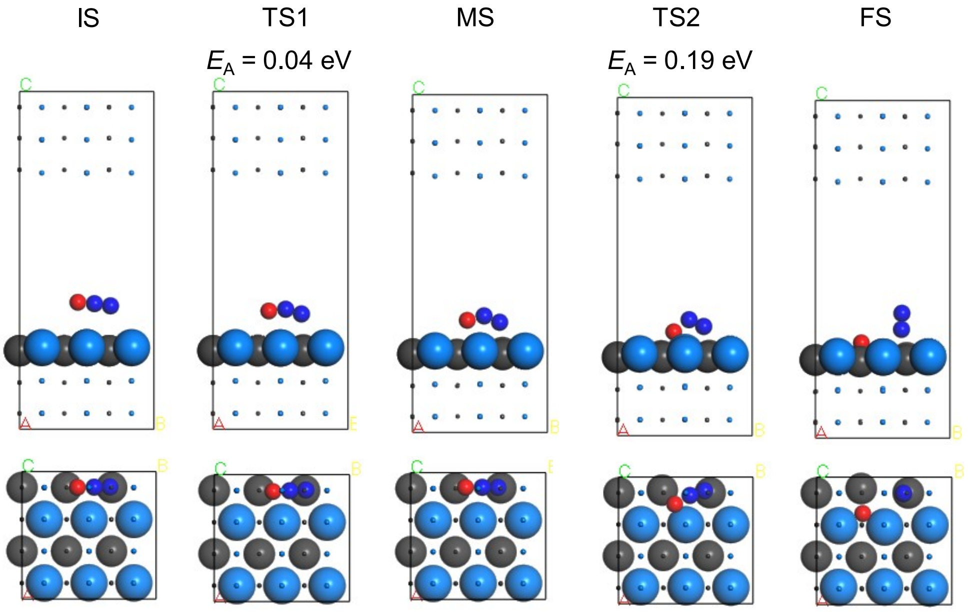

(e) N_2O decomposition over In-terminated PdIn(120)

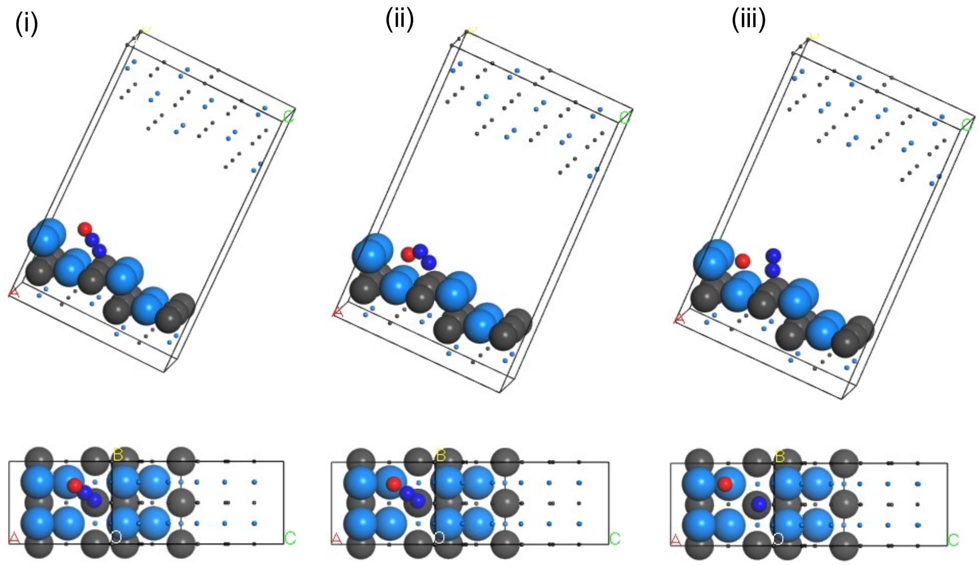

Figure S13. Structures of adsorbates (IS, MS, and FS: initial, intermediate, and final states, respectively) and the corresponding transition states (TS) during N_2O decomposition over (a) Pd(111), (b) Pd(100), (c) Pd(511), (d) PdIn(110), and (e) In-terminated PdIn(120) surfaces. For clarity, metal atoms in the sub-surface region are shown as small dots. For (e), structure (i) (unoptimized) was changed to (ii) during geometry optimization. TS calculation between structures (ii) and (iii) gave a very low E_A less than 0.1 kJmol^{-1}.

(a) N_2 formation over Pd(111)

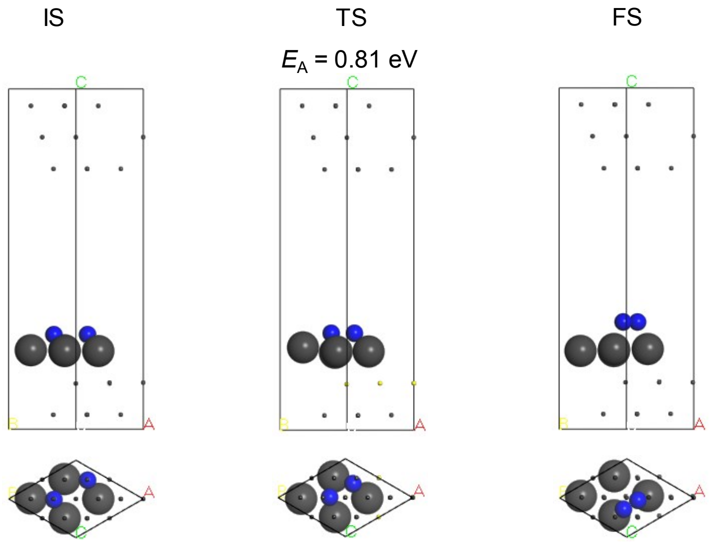

(b) N_2 formation over Pd(111)

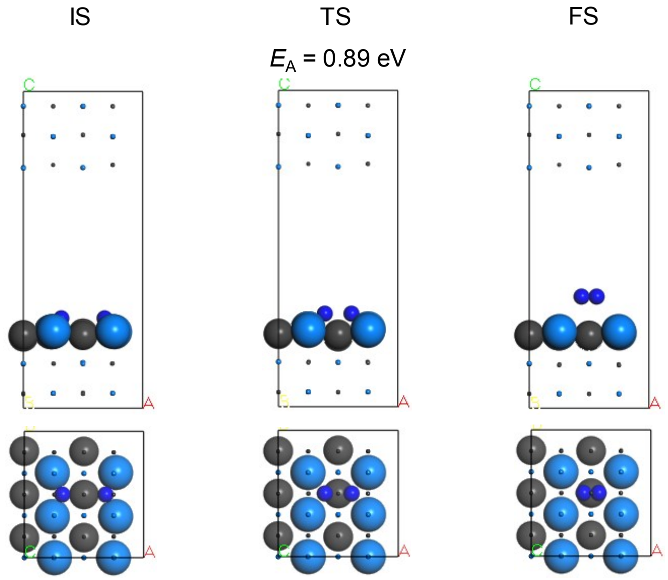

(c) CO oxidation over Pd(111)

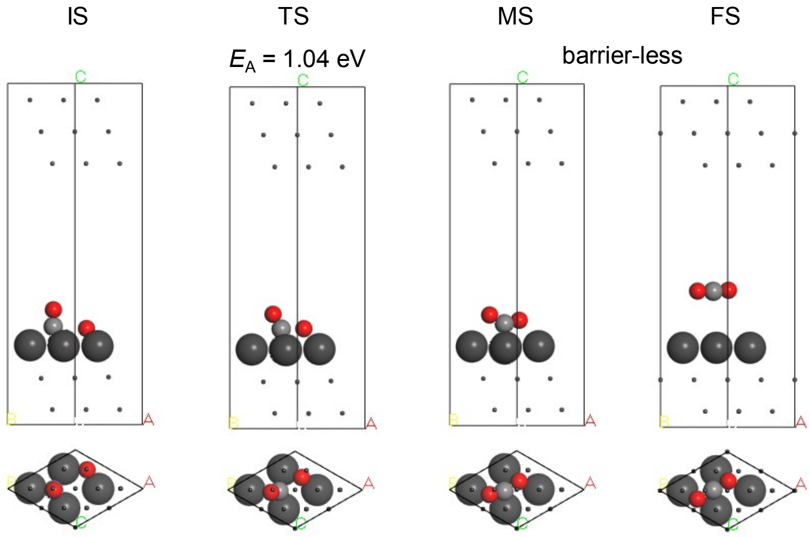

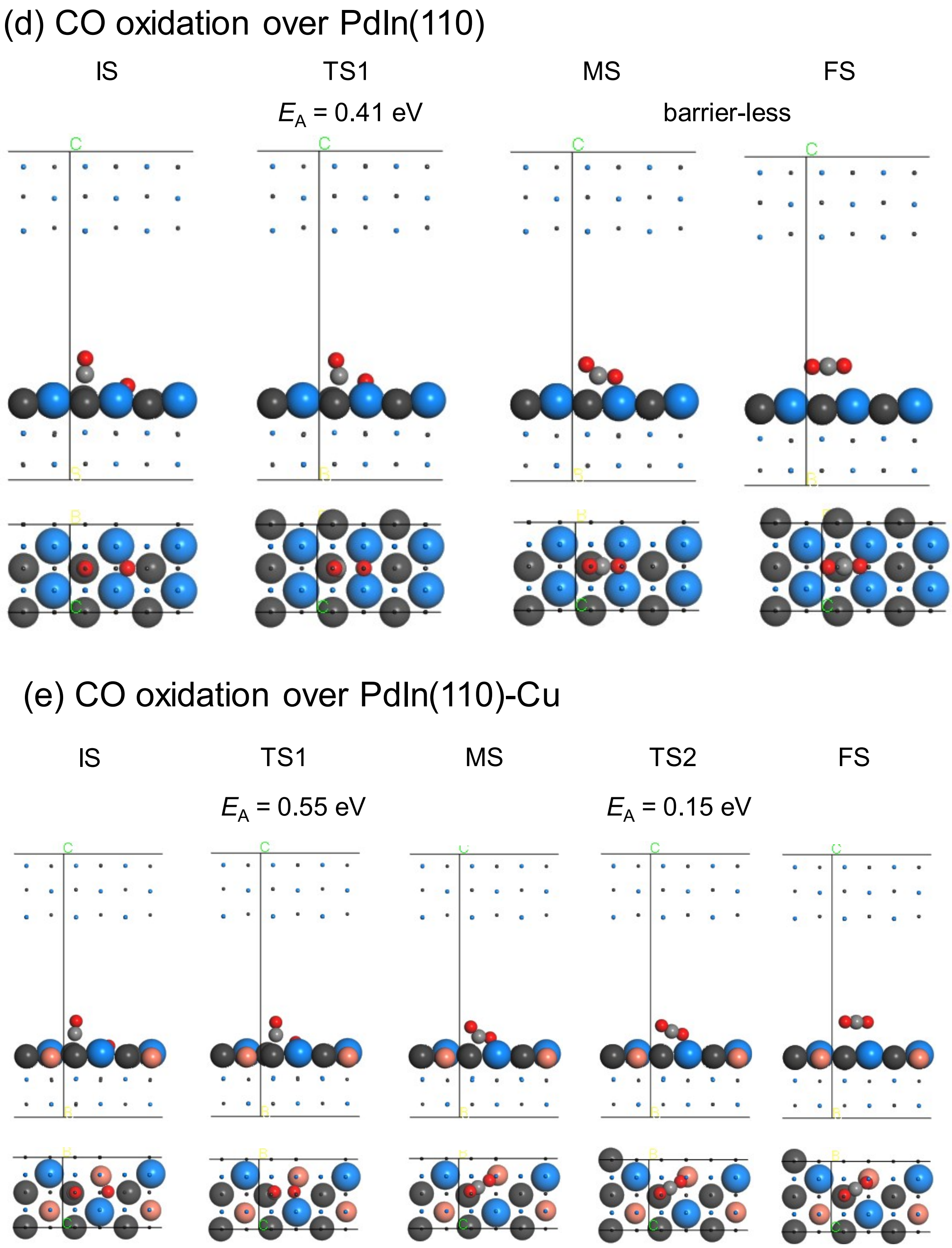

Figure S14. Structures of adsorbates and the corresponding transition states during N_2 formation over (a) Pd(111) and (b) PdIn(110) and CO oxidation over (c) Pd(111), (d) PdIn(110), and (e) Cu-substituted PdIn(110). For clarity, metal atoms in the sub-surface region are shown as small dots. For (c) and (d), an unoptimized MS structure was used for TS calculation between IS and MS (geometry optimization of MS gave the FS structure).

(a) NO dissociation over Pd(100)

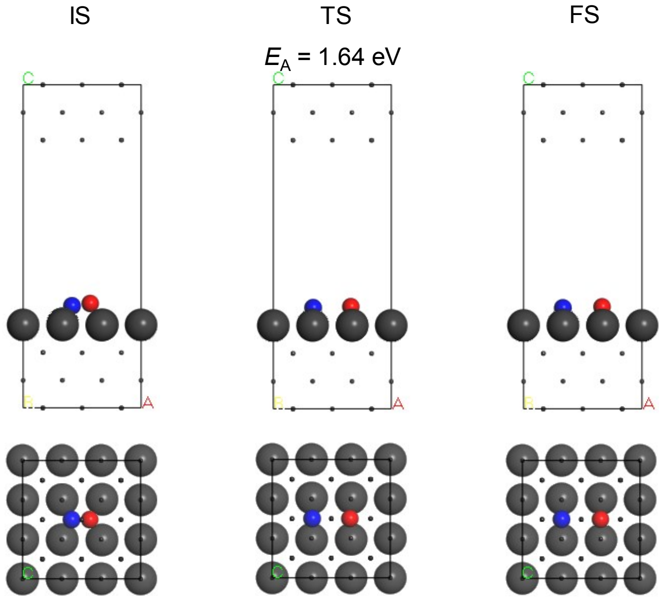

(b) NO dissociation over Pd(511)

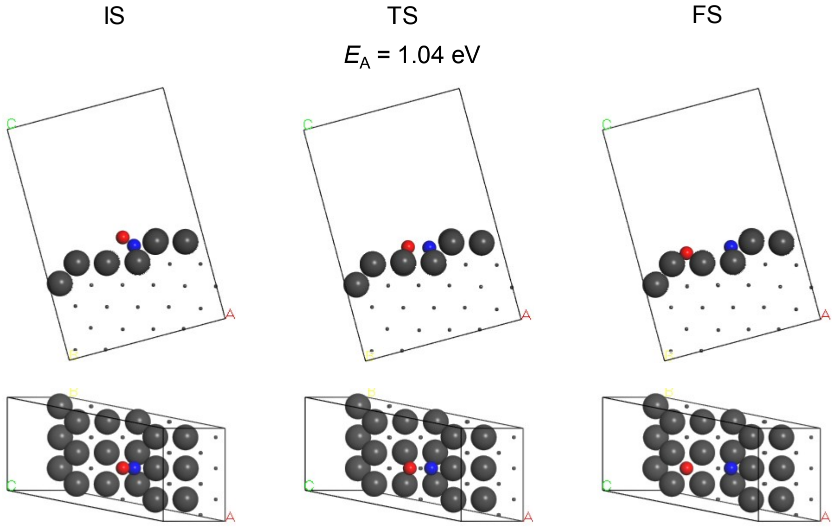

(c) NO dissociation over PdIn(110)

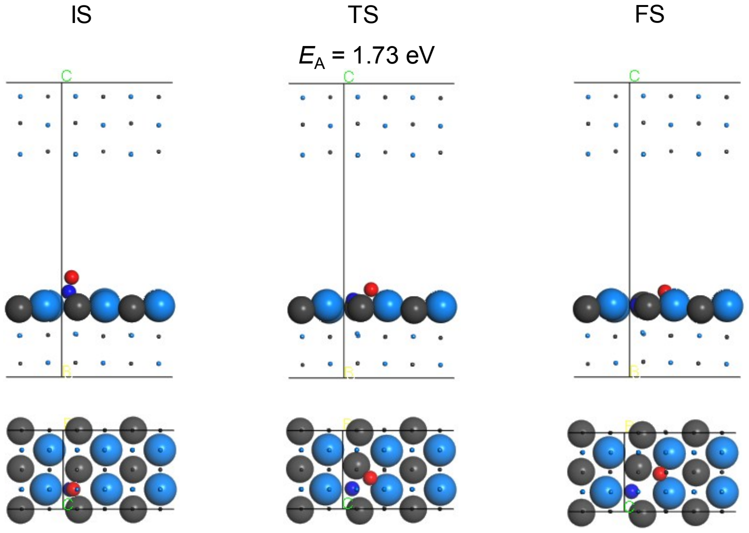

(d) NO dissociation over PdIn(120)

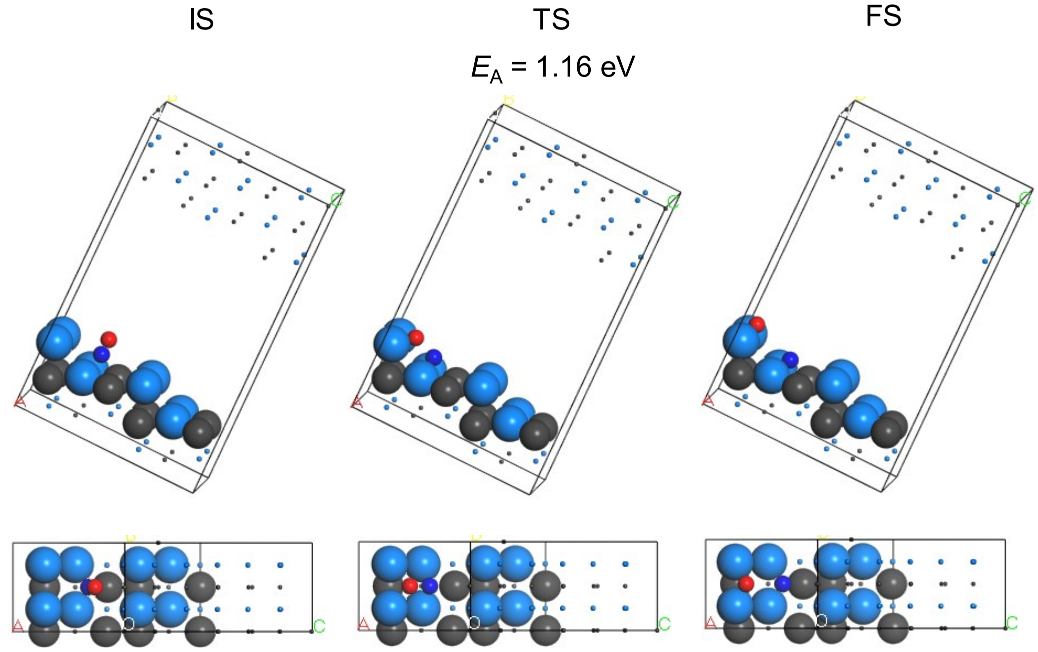

(e) NO dissociation over PdIn(120)–Cu

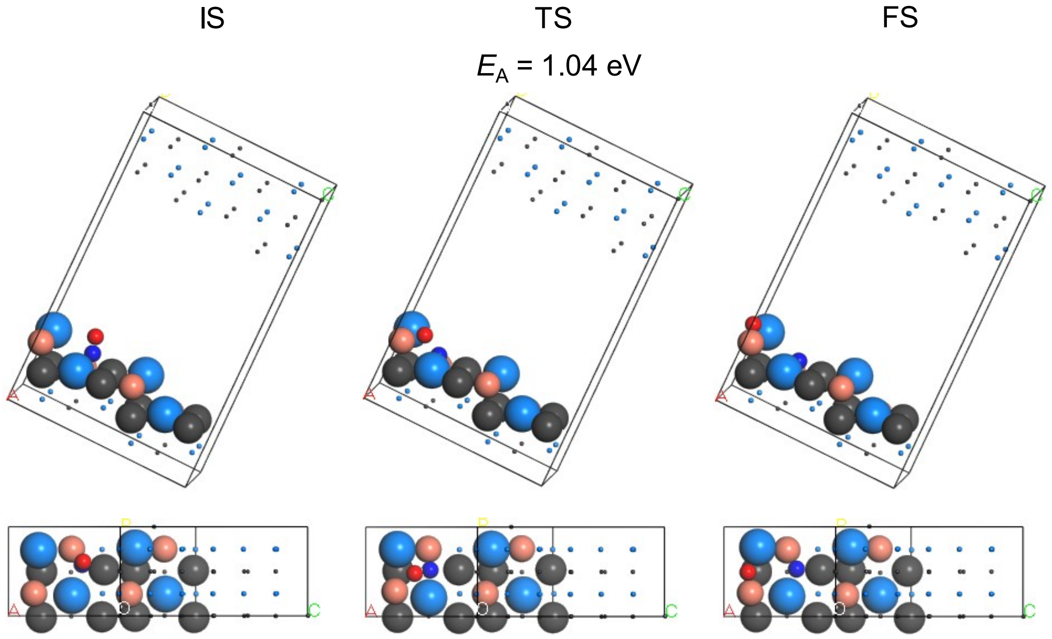

Figure S15. Structures of adsorbates and the corresponding transition states during NO dissociation over (a) Pd(100) and (b) Pd(511), (c) PdIn(110), (d) PdIn(120), and (e) Cu-substituted PdIn(120). For clarity, metal atoms in the sub-surface region are shown as small dots.

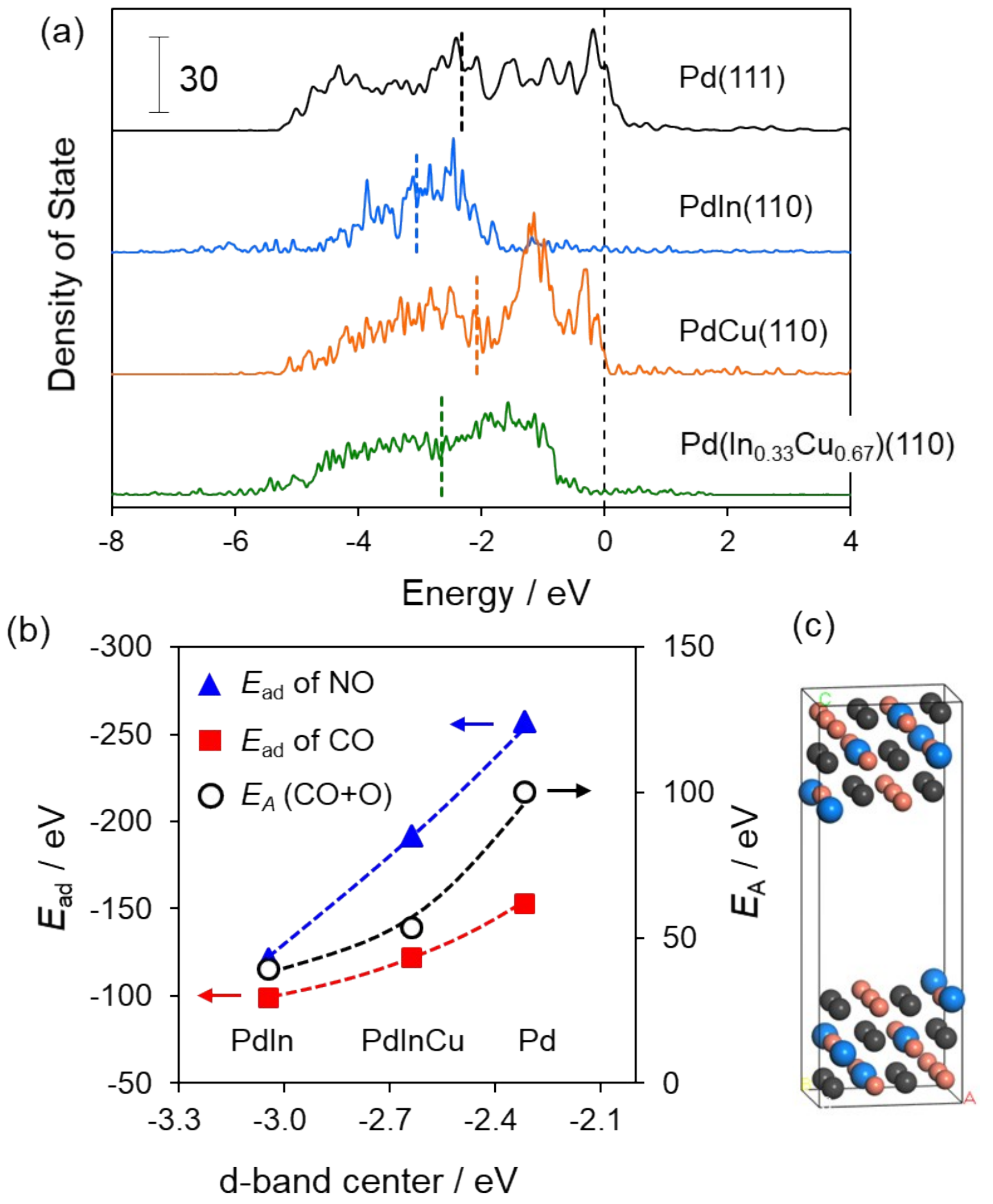

Figure S16. (a) Projected density of states (DOS) of 4d orbitals on Pd(111), PdIn(110), PdCu(110), and Pd($In_{0.33}Cu_{0.67}$)(110) surfaces. The vertical dashed and the bold dotted lines indicate the Fermi level and the position of d-band center, respectively. (b) Correlations between the position of d-band center and E_{ad} of NO or CO and E_A of CO oxidation. (c) Slab model of Pd($In_{0.33}Cu_{0.67}$)(110) used for DOS calculation.

Kinetic Analysis

Considering a Langmuir-Hinshelwood type mechanism for the NO–CO reaction, the reaction steps are described as follows:

$NO + \sigma \rightleftarrows NO \cdot \sigma$ (1): NO adsorption

$CO + \sigma \rightleftarrows CO \cdot \sigma$ (2): CO adsorption

$NO \cdot \sigma + \sigma \rightleftarrows N \cdot \sigma + O \cdot \sigma$ (3): NO dissociation

$2N \cdot \sigma \rightarrow N_2 + \sigma$ (4): N_2 formation

$CO \cdot \sigma + O \cdot \sigma \rightarrow CO_2 + 2\sigma$ (5): CO oxidation (CO_2 formation)

$N \cdot \sigma + NO \cdot \sigma \rightleftarrows N_2O \cdot \sigma + \sigma$ (6): N_2O formation

$N_2O \cdot \sigma \rightleftarrows N_2 + O \cdot \sigma$ (7): N_2O decomposition

$N_2O \cdot \sigma \rightleftarrows N_2O + \sigma$ (8): N_2O desorption

where, σ indicates an adsorption site. The steps (1) ~ (5) are identical to those considered in the conventional kinetic models for NO–CO reaction over Pd and Rh catalysts. The modified points are that N_2O is once formed as an adsorbate (6) and that N_2O decomposition and sorption are considered (7, 8), which are the crucial factors to determine N_2 selectivity in the present system.

We here considered an approximation that for steps (4) and (5), reverse reaction can be ignored under an atmospheric pressure condition. This approximation was supported also by DFT calculation, in which the reaction barriers of the reverse reactions ($E_A + \Delta E$) are much higher than those of the forward reactions (E_A, Table #). Other steps can be regarded to be in equilibrium except the rate-determining step. Therefore, the equilibrium constants are generally defined as follows:

$$K_1 = \theta_{NO}/P_{NO}(1-\theta)$$

$$K_2 = \theta_{CO}/P_{CO}(1-\theta)$$

$$K_3 = \theta_N\theta_O/\theta_{NO}(1-\theta)$$

$$K_6 = \theta_{N_2O}(1-\theta)/\theta_{NO}\theta_N$$

$$K_7 = P_{N_2}\theta_O/\theta_{N_2O}$$

$$K_8 = P_{N_2O}(1-\theta)/\theta_{N_2O}$$

where, P_X, θ_X, and $(1-\theta)$ are the partial pressure of *X*, coverage of *X*, and percentage of vacant site: $1-(\theta_{NO}+\theta_{CO}+\theta_N+\theta_O+\theta_{N_2O})$, respectively.

Assuming that NO adsorption (1) is the rate determining step, the overall reaction rate can be expressed using a rate constant *k* as follows:

$$r = kP_{NO}(1-\theta)$$

Here, $(1-\theta)$ is expressed using the equilibrium constants and P_X as follows:

$$1-\theta = \frac{1}{1+K_2P_{CO}+\sqrt{K_3{K_6}^{-1}K_7{P_{N_2}}^{-1}}\left({K_7}^{-1}P_{N_2}+{K_3}^{-1}{K_8}^{-1}P_{N_2O}\right)+{K_8}^{-1}P_{N_2O}\left(K_7{P_{N_2}}^{-1}+1\right)}$$

Based on these, the overall reaction rate can be descried as follows:

$$r = \frac{kP_{NO}}{1+K_2P_{CO}+\sqrt{K_3{K_6}^{-1}K_7{P_{N_2}}^{-1}}\left({K_7}^{-1}P_{N_2}+{K_3}^{-1}{K_8}^{-1}P_{N_2O}\right)+{K_8}^{-1}P_{N_2O}\left(K_7{P_{N_2}}^{-1}+1\right)}$$

This equation indicates the first-order dependence of *r* on P_{NO} and that the reaction order of P_{CO} ranges from −1 to 0, respectively. This does not agree with the experimental results.

Assuming that CO adsorption (2) is the rate determining step, the overall reaction rate can be expressed using a rate constant *k* as follows:

$$r = kP_{CO}(1-\theta)$$

This equation is described using the equilibrium constants and P_X as follows:

$$r = \frac{kP_{CO}}{1+K_1P_{NO}+{K_5}^{-1}{K_7}^{-1}K_8P_{N_2}{P_{N_2O}}^{-1}P_{CO_2}+{K_8}^{-1}P_{N_2O}\left({K_1}^{-1}{K_6}^{-1}{P_{NO}}^{-1}+K_7{P_{N_2}}^{-1}+1\right)}$$

Here, we temporally used $K_5 = P_{CO_2}(1-\theta)^2/\theta_{CO}\theta_O$ as the equilibrium constant of the step (5) to solve the rate equation. Considering that the rate of forward reaction of step (5) is much faster than the reverse reaction, we can introduce an approximation, $K_5^{-1} \ll 1$.

Therefore, the rate equation can be simplified as follows:

$$r = \frac{kP_{CO}}{1 + K_1 P_{NO} + K_8^{-1} P_{N_2O}\left(K_1^{-1} K_6^{-1} P_{NO}^{-1} + K_7 P_{N_2}^{-1} + 1\right)}$$

This equation indicates the first-order dependence of *r* on P_{CO} and that the reaction order of P_{NO} ranges from −1 to 1, respectively. This does not agree with the experimental results.

Next, we assume that NO dissociation (3) is the rate determining step, the overall reaction rate can be expressed using a rate constant *k* as follows:

$$r = k\theta_{NO}(1-\theta)$$

This equation is described using the equilibrium constants and P_X as follows:

$$r = \frac{kK_1 P_{NO}}{\left\{1 + K_1 P_{NO} + K_2 P_{CO} + (K_1 K_6 K_8)^{-1} P_{N_2O} P_{NO}^{-1} + K_8^{-1} P_{N_2O}\left(K_7 P_{N_2}^{-1} + 1\right)\right\}^2}$$

This equation indicates that the reaction orders of P_{NO} and P_{CO} range from −1 to 3 and from −2 to 0, respectively. This does not agree with the experimental results of positive orders of P_{CO}.

Assuming that N_2 formation (4) is the rate determining step, the overall reaction rate can be expressed using a rate constant *k* as follows:

$$r = k\theta_N^{\,2}$$

This equation is described using the equilibrium constants and P_X as follows:

$$r = k(K_1 K_6 K_8)^{-2} P_{N_2O}^{\,2} P_{NO}^{-2} (1-\theta)^{-2}$$

$$r=\frac{k(K_1K_6K_8)^{-2}P_{N_2O}{}^2P_{NO}{}^{-2}}{\left\{1+K_1P_{NO}+K_2P_{CO}+(K_1K_6K_8)^{-1}P_{N_2O}P_{NO}{}^{-1}+K_1{}^2K_3K_6K_8P_{N_2O}{}^{-1}P_{NO}{}^2+K_8{}^{-1}P_{N_2O}\right\}^2}$$

This equation indicates that the reaction orders of P_{NO} and P_{CO} range from −6 to 0 and from −2 to 0, respectively, being inconsistent with the experimental positive orders.

Then, we assume that CO oxidation (5) is the rate determining step, affording the overall reaction rate expressed as follows:

$$r=k\theta_{CO}\theta_O$$

$$r=kK_2K_7K_8{}^{-1}P_{N_2}{}^{-1}P_{N_2O}P_{CO}(1-\theta)^{-2}$$

$$r=\frac{kK_2K_7K_8{}^{-1}P_{N_2}{}^{-1}P_{N_2O}P_{CO}}{\left\{1+K_1P_{NO}+K_2P_{CO}+(K_1K_6K_8)^{-1}P_{N_2O}P_{NO}{}^{-1}+K_1{}^2K_3K_6K_8P_{N_2O}{}^{-1}P_{NO}{}^2+K_8{}^{-1}P_{N_2O}\right\}^2}$$

The reaction orders of P_{NO} and P_{CO} can range from −4 to 2 and from −1 to 1, respectively, which is well consistent with the experimental positive orders.

When N_2O formation (6) is assumed as the rate determining step, the overall reaction rate is expressed as follows:

$$r=k\theta_N\theta_{NO}$$

$$r=kK_1{}^2K_3K_7{}^{-1}K_8P_{N_2}P_{N_2O}{}^{-1}P_{NO}{}^2(1-\theta)^{-2}$$

$$r=\frac{kK_1{}^2K_3K_7{}^{-1}K_8P_{N_2}P_{N_2O}{}^{-1}P_{NO}{}^2}{\left\{1+K_1P_{NO}+K_2P_{CO}+K_1K_3K_7{}^{-1}K_8P_{N_2}P_{N_2O}{}^{-1}P_{NO}+K_8{}^{-1}P_{N_2O}\left(K_7P_{N_2}{}^{-1}+1\right)\right\}^2}$$

The reaction orders of P_{NO} and P_{CO} range from −2 to 0 and from −1 to 0, respectively, which is inconsistent with the experimental positive orders.

When N_2O decomposition (7) is assumed as the rate determining step, the overall reaction rate is expressed as follows:

$$r = k\theta_{N_2O}$$

$$r = kK_8^{-1}P_{N_2O}(1-\theta)$$

$$r = \frac{kK_8^{-1}P_{N_2O}}{1 + K_1P_{NO} + K_2P_{CO} + (K_1K_6K_8)^{-1}P_{N_2O}P_{NO}^{-1} + K_1^2K_3K_6K_8P_{N_2O}^{-1}P_{NO}^2 + K_8^{-1}P_{N_2O}}$$

The reaction orders of P_{NO} and P_{CO} range from −2 to 0 and from −1 to 0, respectively, which is inconsistent with the experimental positive orders.

When N_2O desorption (8) is assumed as the rate determining step, the overall reaction rate is expressed as follows:

$$r = k\theta_{N_2O}$$

$$r = kK_1K_3^{0.5}K_6^{0.5}K_7^{-0.5}P_{N_2}^{0.5}P_{NO}(1-\theta)$$

$$r = \frac{kK_1\sqrt{K_3K_6K_7^{-1}P_{N_2}}P_{NO}}{1 + K_1P_{NO} + K_2P_{CO} + \sqrt{K_3K_6K_7P_{N_2}}\left(K_6^{-1}K_7^{-1} + K_1P_{N_2}^{-1}P_{NO} + K_1K_7^{-1}P_{NO}\right)}$$

The reaction orders of P_{NO} and P_{CO} range from −1 to 1 and from −1 to 0, respectively, which is inconsistent with the experimental positive order of P_{CO}.

Thus, assuming CO oxidation (5) as the rate-determining step exclusively gave reaction orders consistent with the experiment. On the basis of this result, we concluded that the rate-determining step of NO–CO reaction over Pd-based catalysts is CO oxidation.
